# Biochemical Profiling for Antioxidant and Therapeutic Potential of Pakistani Chickpea (*Cicer arietinum* L.) Genetic Resource

**DOI:** 10.3389/fpls.2021.663623

**Published:** 2021-04-13

**Authors:** Saima Jameel, Amjad Hameed, Tariq Mahmud Shah

**Affiliations:** Nuclear Institute for Agriculture and Biology College, Pakistan Institute of Engineering and Applied Sciences, Faisalabad, Pakistan

**Keywords:** desi, kabuli, hydrolytic enzymes, alpha amylase inhibition, anti-inflammatory

## Abstract

In Pakistan, chickpeas (*Cicer arietinum* L.) are the largest grown legume crops, especially in desert areas. Along with an excellent source of nutrition, chickpea seeds have discernible medicinal and antioxidant characteristics. The diverse set of 90 chickpea genotypes (66 desi and 24 kabuli) were collected from different research zones in Pakistan, and seed flour was used for biochemical profiling. Genotypes were significantly different (Tukey HSD test, *P* < 0.05) for the traits under investigation. In non-enzymatic antioxidants, highest seed total phenolic contents (TPC) (34725 ± 275 μM/g s. wt.) was found in CM-98 (desi), ascorbic acid (AsA) (69.23 ± 2.25 μg/g s. wt.) in WH-3 (desi), and total flavonoid content (TFC) (394.98 ± 13.06 μg/mL sample) was detected in WH-11 (desi). In the class of enzymatic antioxidants, the highest seed ascorbate peroxidase (APX) (1680 ± 40 Units/g s. wt.) was detected in Tamman-2013 (kabuli), peroxidases (POD) (2564.10 ± 233.10 Units/g s. wt.) activity in CM1235/08 (desi), and superoxide dismutase (SOD) (279.76 ± 50 Units/g s. wt.) was detected in CH24/11 (desi). Highest seed catalase activity (CAT) (893 ± 50 Units/g s. wt.) and proline content (272.50 ± 20.82 μg/g s. wt.) was detected in an ICC-4951 (desi). In hydrolytic enzymes, the highest activity of esterase (37.05 μM/min/g s. wt) was found in, CH56/09(Kabuli), protease (11080 ± 10 Units/g s. wt.) in Karak-2 (desi), and α-amylase (213.02 ± 3.20 mg/g s. wt.) was observed in CH74/08 (kabuli). In other biochemical parameters, the highest seed total oxidant status (TOS) (356 ± 17.50 μM/g s. wt.) was detected in CM3457/91 (desi); malondialdehyde (MDA) content (295.74 ± 3.097 uM/g s. wt.) was observed in CM-2008 (kabuli), and total antioxidant capacity (TAC) (8.36 ± 0.082 μM/g s. wt.) was found in CM-72 (desi). In case of pigment analysis, Sheenghar-2000 (desi) depicted highest lycopene (12.579 ± 0.313 μg/g s. wt.) and total carotenoids (58.430.23 ± 0.569 μg/g s. wt.) contents. For seed therapeutic potential, the highest seed α-amylase inhibition (82.33 ± 8.06%) was observed in CM-88 (desi), while WH-1, WH-6, and ICCV-96030 (desi) depicted the highest value for seed anti-inflammatory potential (78.88 ± 0.55%). Genotypes with the highest antioxidant and therapeutic potential can be utilized as a natural antioxidant source and in breeding programs aimed at improving these traits in new breeding lines.

## Introduction

Oxidative stress, induced by Reactive oxygen species (ROS), plays an imperative role in the development and progression of numerous life-threatening degenerative diseases in the body, i.e., cardiovascular disease, cancer, diabetes etc. (Glasauer and Chandel, 2013; Kabel, 2014; Kasote et al., 2015; Di Meo et al., 2016; Ahmed and Mohammed, 2020; Taha et al., 2021). Antioxidants are compounds that have the ability, even in minor concentrations, to avert and delay the oxidation of easily oxidizable substances (Brewer, 2011; Sen and Chakraborty, 2011). According to a WHO report, the global population of people with diabetes will increase from 592 to 2035 billion in the near future. Along with diabetes and cancer, inflammatory diseases have increased tremendously, causing millions of deaths throughout the world in recent years (Pradhan et al., 2021a). Unfortunately, several adverse effects, such as toxicity, metabolic impairments, and drug tolerance are strongly associated with chemotherapeutic, anti-inflammatory, and anti-diabetic drugs (Pradhan et al., 2021b). A promising way to counteract the undesirable effects of reactive oxygen species (ROS) in the body, however, is with the supplementation of food rich in antioxidants and that possess high therapeutic capacity (Kasote et al., 2013; Cammisotto et al., 2021). Antioxidants are normally classified into two main groups−natural and synthetic antioxidants (Kumar et al., 2020; Rios-Mera et al., 2021). The synthetic group of antioxidants have been used as food additives for an extended period of time, but are highly restricted due to their genotoxic effects (Anwar et al., 2018; Xu et al., 2021). Consequently, the natural antioxidants obtained from botanical sources, especially edible plants, have acquired significant attention in the management of oxidative stress-related illnesses (Zia-Ul-Haq et al., 2008; Vaiserman et al., 2020; Badr et al., 2021; Mahbub et al., 2021). Plants have long been a cheap and rich source of exogenous antioxidant supplements. It is assumed that among all the plant species, two-thirds of all plant species across the world have excellent antioxidant and therapeutic potentials (Krishnaiah et al., 2011; Jamshidi-Kia et al., 2020; Xu et al., 2021). The idea of exogenous plant-based antioxidants gained importance after the discovery and isolation of ascorbic acid from different plant parts (Szent-Györgyi, 1963). Epidemiologists recommend the intake of more legumes (chickpea, soybean, lentil, cowpea etc.) and fruits because they are rich in natural antioxidants, moreover, it may also help in alleviating any potential nutritional gap in the body caused by unhealthy and low quality food consumption (Diniyah et al., 2020; Swallah et al., 2020; Matemu et al., 2021). Currently, several *in vitro* methods are being used to determine the different antioxidants found in plants (Alam et al., 2013).

Chickpea (Cicer arietinum L.) is the 3rd most imperative cool-season grain legume crop after the common bean (Phaseolus vulgaris L.) and field pea (Pisum sativum L.), and is the second most grown crop by poor farmers, primarily in the arid and semi-arid regions of Pakistan (Kinfe et al., 2015; Jan et al., 2020; Rafiq et al., 2020). It is commonly classed into two different market classes; the desi type (small seed size, dark in color) and the kabuli type (larger seed size, light color) and covers about 85% and 15% of the global chickpea production area, respectively (Kabuo et al., 2015; Ugandhar et al., 2018). After India and Turkey, Pakistan is the third-largest commercial producer of chickpeas with a 7% share in global production (Thudi et al., 2016; Maurya and Kumar, 2018). In Pakistan, out of the total area used for pulse production, chickpeas cover 73% of the area and shares 76% of the gross production (Ullah et al., 2020). It contributes about 4.7% to the domestic economy of the country with an annual production of 760,000 million tons, from an area of 1.094 million hectares (Chandio et al., 2016). Chickpea seeds are a principal and inexpensive source of highly digestible nutrients such as proteins, carbohydrates, vitamins, fibers, minerals, and essential amino acids especially for people in developing countries (Dhankhar et al., 2019). It plays a crucial role in ensuring good nutrition and provides food security as its flour contains a higher protein, ash, mineral, and fat content than wheat flour (Rachwa-Rosiak et al., 2015). Besides being an excellent source of fundamental nutrients, chickpea seeds contain a diverse range of certain bioactive compounds that show high antioxidant capacity, anti-diabetic, and anti- inflammatory properties etc., in this way it helps to improve health by decreasing the incidence of diseases (Garzón-Tiznado et al., 2012; Gupta et al., 2017; Cid-Gallegos et al., 2020; Faridy et al., 2020; Ramadhani et al., 2020; Mahbub et al., 2021). Chickpea comprises a broad variety of polyphenols such as flavone glycosides, flavonols, polymeric pro anthocyanidins, and oligomers. These polyphenols are adept at intercepting singlet oxygen, decreasing oxygen concentration, and deterring first chain instigation by quenching primary radicals (Zia-Ul-Haq et al., 2008).

At present there is a need to shift the agriculture system away from producing larger quantities of food, to producing more nutritious and high-quality food crops instead (Garg et al., 2018). To date, limited research is available on chickpea seed-based antioxidant profiles and pharmacological properties, and grain composition has been badly compromised over grain quantity in chickpea breeding programs (Zia-Ul-Haq et al., 2007; Zia-Ul-Haq et al., 2008; Garzón-Tiznado et al., 2012). The main interest of the study was the seed-based evaluation of the Pakistani chickpea genetic resource, including landraces, approved varieties, wild crosses, advance lines, mutants for high antioxidant capacity, and their therapeutic potentials. Thus, comprehensive seed antioxidants, in vitro anti-inflammatory, and in vitro anti-diabetic analyses were performed.

## Materials and Methods

A diverse set of 90 chickpea genetic resources, comprising 66 desi and 24 kabuli types with different genetic backgrounds, were used in this study. The mature dry seeds of the germplasm, harvested between 2018 and 2019, were collected from different research stations in Pakistan for the evaluation of their seed-based antioxidants, anti-diabetic, and anti-inflammatory capacity. All genotypes were cataloged for pedigree information, tolerance against biotic and abiotic stresses, and other salient agronomic features (Table 1). Foreign material and damaged seeds were removed, and the samples were stored at room temperature for 45 days in cotton bags to attain equal levels of moisture content. The seed compositional analysis was carried out at Marker Assisted Breeding Lab-1 Plant Breeding and Genetics Division, Nuclear Institute for Agriculture and Biology (NIAB), Faisalabad, Pakistan.

**TABLE 1:** Chickpea genotypes along with known traits, used in the study.

Sr#	Variety	Year of Release	Institution	Botanical Name	Parentage/pedigree	Average Yield (kg/ha)	Type	Traits of importance
1	CM-72	1982	NIAB, FSD	*C. arietinum*	6153 at 150Gy	1205	Desi	High yielding, small seeded, tolerant to ascochyta blight
2	C-44	1982	AARI, FSD	*C. arietinum*	Selection	1500-1600	Desi	High yielding, bold seeded, tolerant to ascochyta blight, susceptible to iron chlorosis.
3	Paidar-91	1992	AARI, FD	*C. arietinum*	Local cross: C235 × ILC191-1	1700-1800	Desi	High yielding, medium seeded, tolerant to ascochyta blight.
4	NIFA-88	1992	NIFA, Peshawar	*C. arietinum*	Local Mutant: 6153 (10 kr)	1500-1600	Desi	High yielding small seeded, tolerant to ascochyta blight.
5	Karak-1	1992	GRS, Karak	*C. arietinum*	Local selection	920-1280	Desi	Small seeded, drought tolerant, tolerant to blight
6	Punjab-91	1992	AARI, FSD	*C. arietinum*	Local cross: NEC I 38-2/RC 32	1500-1600	Desi	High yielding, bold seeded, tolerant to Ascochyta blight, Late maturity & susceptible to shattering.
7	CM88	1994	NIAB, FSD	*C. arietinum*	Local Mutant C 727 (10 kr)	1700-1900	Desi	High yielding, blight and wilt resistant, wider adaptability, semi erect,
8	NIFA-95	1996	NIFA, Peshawar	*C. arietinum*	Local Mutant 6153 (10 kr)	1500-1600	Desi	Tolerant to blight and storage insects
9	CM98	1998	NIAB, FSD	*C. arietinum*	Local Mutant K 850 (300 Gy)	1600-1900	Desi	High yielding, blight and wilt resistant, wider adaptability
10	Bittle-98	1998	AARI, FSD	*C. arietinum*	(C44 × C44 × C87)	1500-1600	Desi	High yielding, bold seeded, tolerant to ascochyta blight. Resistant to iron chlorosis.
11	Sheenghar-2000	2000	GRS, Karak	*C. arietinum*	Local selection	1600-1700	Desi	Drought and blight tolerant, bold seeded, high yielding
12	Punjab-2000	2000	AARI, FSD	*C. arietinum*	Local cross C 87/C 44	1700-1800	Desi	High yielding, bold seeded, tolerant to ascochyta blight, resistant to shattering.
13	Balkasar2000	2000	BARI, Chakwal	*C. arietinum*	ILC 5928/ {(ILC5928/ILC72) (ILC3856/E-100 YM)}	1500-1600	Desi	High yielding, medium seeded, tolerant to ascochyta blight, suitable for cultivation in Pothohar region.
14	Wanhar	2000	BARI, Chakwal	*C. arietinum*	Exotic selection: ILC 5928/ {(ILC5928/ILC72) (ILC3856/E100YM)}	1600-1800	Desi	High yielding, medium seeded, tolerant to ascochyta blight, suitable for cultivation in Pothohar region.
15	Dashat 98	2003	NARC, ISLD	*C. arietinum*	(C-44 × ICC7770)	1500-1600	Desi	High yielding medium seeded, resistant to ascochyta blight, suitable for cultivation in Pothohar region.
16	Parbat 98	2003	NARC, ISLD	*C. arietinum*	Local cross ICC 11514/ILC 3279	1500-1600	Desi	Higher yielder, medium seeded, resistant to ascochyta blight, suitable for cultivation in Pothohar region.
17	Karak-2	2003	GRS, Karak	*C. arietinum*	Local selection	920-1200	Desi	Drought tolerant, medium seed size
18	Thal-2006	2006	AZRI, Bhakkar	*C. arietinum*	(CM82/CM87 × C-44)	2096	Desi	Bold seeded, drought and blight tolerant, highly responsive to irrigation.
19	Punab2008	2008	AARI, FSD	*C. arietinum*	(90065 × ICC 12231)	2343	Desi	High yielding, wilt resistant and tolerant to blight
20	Bhakhar-2011	2011	AZRI, Bhakkar	*C. arietinum*	(90243 × Paidar-91)	2312	Desi	Bold seeded, drought and blight tolerant, wilt resistant
21	NIAB-CH2016	2016	NIAB, FSD	*C. arietinum*	(96052 × pb2000)	1929	Desi	High yielding, wilt resistant and tolerant to blight
22	Bittle-2016	2016	PRI, AARI, FSD	*C. arietinum*	Punab-2000 × ICC-5127	3993	Desi	High yielding, wilt resistant and tolerant to blight
23	Karak 3	2003	ARS, Karak NARC	*C. arietinum*	Local selection	950-1230	Desi	High yielding, medium duration, Small plant
24	NIAB-CH104	2019	NIAB, FSD	*C. arietinum*	Pusa-329XCM3444/92	2055	Desi	High Yielding, Semi erect, moderately resistant to wilt and blight, good for Barani and irrigated area
25	CH28/07	AL	NIAB, FSD	*C. arietinum*	CH31/99pb2008		Desi	
26	CH40/09	AL	NIAB,FSD	*C. arietinum*	VBRC61X pb2008		Desi	
27	CH49/09	AL	NIAB,FSD	*C. arietinum*	CM1198/93X pb2008		Desi	
28	CH39/08	AL	NIAB,FSD	*C. arietinum*	CH19/99xCM1655/88		Desi	
29	CH32/10	AL	NIAB,FSD	*C. arietinum*	(CM98xreti1-1-1)x97086		Desi	
30	CH35/10	AL	NIAB,FSD	*C. arietinum*	(CM98xreti1-5-2)x138/03		Desi	
31	CH2/11	AL	NIAB,FSD	*C. arietinum*	CM72xPb2003		Desi	
32	CH10/11	AL	NIAB,FSD	*C. arietinum*	CM551/05XCH23/00		Desi	
33	CH13/11	AL	NIAB,FSD	*C. arietinum*	Pb91x01067		Desi	
34	CH24/11	AL	NIAB,FSD	*C. arietinum*	CH1/03xPb2008		Desi	
35	CH39/11	AL	NIAB,FSD	*C. arietinum*	CH38/04XB8/03		Desi	
36	CH19/10	AL	NIAB,FSD	*C. arietinum*	CM601/03x97086		Desi	
37	CH16/06	AL	NIAB,FSD	*C. arietinum*	P1-13xPusa329		Desi	
38	CH24/07	AL	NIAB,FSD	*C. arietinum*	CH31/99Xpb2008		Desi	
39	CM2984/91	ML	NIAB,FSD	*C. arietinum*			Desi	
40	CM1051/11	ML	NIAB,FSD	*C. arietinum*			Desi	
41	CM1681/8	ML	NIAB,FSD	*C. arietinum*			Desi	
42	CM3384/00	ML	NIAB,FSD	*C. arietinum*			Desi	
43	CM407/13	ML	NIAB,FSD	*C. arietinum*			Desi	
44	CM3444/92	ML	NIAB,FSD	*C. arietinum*			Desi	
45	CM3457/91	ML	NIAB,FSD	*C. arietinum*			Desi	
46	Wild Cross-1	WC	NIAB,FSD	*C. arietinum*	CM98/reti 599072		Desi	
47	Wild Cross -2	WC	NIAB,FSD	*C. arietinum*	CM98/reti 599072		Desi	
48	Wild Cross -3	WC	NIAB,FSD	*C. arietinum*	CM98/reti 599072		Desi	
49	Wild Cross -4	WC	NIAB,FSD	*C. arietinum*	CM98/reti 599072		Desi	
50	Wild Cross -5	WC	NIAB,FSD	*C. arietinum*	CM98/reti 599072		Desi	
51	Wild Cross d-6	WC	NIAB,FSD	*C. arietinum*	Pb-1/reti 599072		Desi	
52	Wild Cross -7	WC	NIAB,FSD	*C. arietinum*	Pb-1/reti 599072		Desi	
53	Wild Cross -8	WC	NIAB,FSD	*C. arietinum*	Pb-1/reti 599072		Desi	
54	Wild Cross -9	WC	NIAB,FSD	*C. arietinum*	Pb-1/reti 599072		Desi	
55	Wild Cross 10	WC	NIAB,FSD	*C. arietinum*	Pb-1/reti 59907		Desi	
56	Wild Cross -11	WC	NIAB,FSD	*C. arietinum*	CM98/reti 599072(2-4-1)/pb-2000		Desi	
57	Wild Cross 12	WC	NIAB,FSD	*C. arietinum*	CM98/reti 599072(1-5-2)/pb-2000		Desi	
58	Wild Cross 13	WC	NIAB,FSD	*C. arietinum*			Desi	
59	Wild Cross -14	WC	NIAB,FSD	*C. arietinum*	BK2011 × ILWC247 (p7)		Desi	
60	Wild Cross -15	WC	NIAB,FSD	*C. arietinum*	BK2011 × ILWC247 (p3)		Desi	
61	Wild Cross -16	WC	NIAB,FSD	*C. arietinum*	5CC109 × ILWC247 (p1)		Desi	
62	Aug-424		UAF	*C. arietinum*			Desi	
63	ICCV-96030	EL	(ICRISAT)	*C. arietinum*	P458 {[(K850 × GW-GW5/7) × (L550 × Gaumuchil916) × (ICC1069 × TCPS50467)]}	730	Desi	Cold-tolerant, Early in flowering/podding
64	ICCV96029	EL	(ICRISAT)	*C. arietinum*	P458 {[(K850 × GW-GW5/7) × (L550 × Gaumuchil916) × (ICC1069 × TCPS50467)]}	302-795	Desi	Cold-tolerant, Early in flowering/podding
65	ICC-4951	1986	(ICRISAT)	*C. arietinum*			Desi	Drought Tolerant, Cold Susceptible
66	ILWC-247	1975		*(C. reticulatum)*			Desi	Tolerance to abiotic and abiotic stresses
67	Noor-91	1992	AARI,FSD	*C. arietinum*	Exotic selection	1500-1600	Kabuli	High yielding, bold seeded, tolerant to ascochyta blight.
68	Lwaghar2000	2000	GRS, Karak	*C. arietinum*	Local selection	1500-1600	Kabuli	Medium bold seeded, Drought and wilt tolerant
69	CM-2000	2000	NIAB, FSD	*C. arietinum*	Local mutant ILC195 (150 Gy gamma rays)	1700-1800	Kabuli	High yielding, tolerant to ascochyta blight, suitable for cultivation in irrigated & rice growing areas.
70	CM2008	2008	NIAB, FSD	*C. arietinum*	Mutant (Punjab-1) EMS 0.2%	1800	Kabuli	Medium seeded, high yielding, wilt resistant and tolerant to blight
71	Noor 2009	2009	AARI, FSD	*C. arietinum*		1700-1800	Kabuli	High yielding, tolerant to Fusarium wilt, suitable for cultivation in irrigated areas
72	Noor 2013	2009	AARI, FSD	*C. arietinum*	K-96033 × K-92029	1920	Kabuli	High yielding, wilt resistant and tolerant to blight
73	Tamaman-13	2013	BARI, Chakwal	*C. arietinum*			Kabuli	High yielding, wilt resistant
74	CH74/08	AL		*C. arietinum*	70022 × CM2008		Kabuli	
75	CH77/08	AL		*C. arietinum*	70022 × CM2008		Kabuli	
76	CH55/09	AL		*C. arietinum*	70022 × CM2008		Kabuli	
77	CH56/09	AL		*C. arietinum*	70022 × PKV2		Kabuli	
78	CH61/09	AL		*C. arietinum*	70022 × CH38/00		Kabuli	
79	BKK-2174	AL		*C. arietinum*			Kabuli	
80	CH63/11	AL		*C. arietinum*	K70009 × CM2008		Kabuli	
81	CH64/11	AL		*C. arietinum*	K70009 × CM2008		Kabuli	
82	CH74/10	AL		C. arietinum	PKV2 × CM86/02		Kabuli	
83	CH68/08	AL		C. arietinum	70022 × CM2008		Kabuli	
84	CH54/07	AL		C. arietinum	92019 × CC98/99		Kabuli	
85	CH60/10	AL		C. arietinum	Noor2009 × CC98/99		Kabuli	
86	CH98/99	AL		C. arietinum	Pb1 Colchicine		Kabuli	
87	CH72/08	AL		C. arietinum	70022 × CM2008		Kabuli	
88	CM1235/08	ML		C. arietinum	70022, 0.2%EMS		Kabuli	
89	CM877/10	ML		C. arietinum	K70009 × 0.2%EMS		Kabuli	
90	Gocke	EL	Turkey	C. arietinum		337	Kabuli	Drought tolerant

*AL, Advanced Line; ML, Mutant Line; WC, Wild Crosses; EL, Exotic Line.*

### Determination of Antioxidant Activities

#### Sample Extraction

A laboratory mini mill grinder was used to grind 20 healthy (4 to 6 g) disease-free seeds into fine powder, and the material was passed through an 80 μm sieve. A sample (0.2 g) was extracted in 2 mL (50 mM) potassium phosphate buffer (pH 7.4). To homogenize the mixture, all the samples were vortexed then centrifuged at 14,462 × g for 10 min at 4°C. For the determination of different biochemical analyses, supernatant was separated and used according to different methods (Khalid and Hameed, 2017). Data were recorded in triplicate for all biochemical parameters.

#### Non-enzymatic Antioxidants

##### Total phenolic content (TPC)

For determination of total phenolic content, a previously defined method (Ainsworth and Gillespie, 2007) was followed, in which Folin-Ciocalteau (FC) reagent was used. For this purpose, 0.5 g seed samples were homogenized in 500 μl ice cold 95% methanol, using an ice-cold mortar and pestle. The samples were then incubated in the dark at room temperature for 48 h. After incubation, samples were centrifuged at 14,462 × g at room temperature for 5 min. After centrifugation the supernatant was taken and TPC was measured by mixing 100 μl with 100 μl of 10% (v/v) F-C reagent, then vortexed thoroughly, and then 800 μl of 700 mM Na_2_CO_3_ was added. The samples were then placed in an incubator for 1 h. The first blank reading was recorded at 765 nm, then a standard curve was prepared by measuring at different gallic acid concentrations (300, 400, 500, 600, 700, and 800 μM/100 μL) and a linear regression equation was calculated. The phenolic content (Gallic acid equivalents) of samples was recorded using the linear regression equation obtained.

##### Ascorbic acid (AsA)

To measure reduced ascorbic acid, the previously described 2,6-dichloroindophenol (DCIP) method (Hameed et al., 2005) was followed. In this method, vitamin C converts a molecule of DCIP into DCIPH_2_ and this conversion can be detected by a decrease in absorbance at 520 nm. A standard curve was prepared using a series of known ascorbic acid concentrations, then a simple linear regression linear equation was used to find the ascorbate concentration in unknown samples.

##### Total flavonoid content (TFC)

The total flavonoid content was determined according to the aluminum chloride colorimetric method (Lin and Tang, 2007). The sample (400 μ + 1.6 mL dH_2_O) was mixed with 0.1 mL of 10% aluminum chloride hexahydrate, 0.1 mL of 1 M potassium acetate, and 2.8 mL of deionized water. After 40-min incubation at room temperature, the absorbance of the reaction mixture was measured by spectrophotometer at 415 nm. Rutin was used as a standard (concentration range: 0.005 to 0.1 mg/mL), and the total flavonoid content was expressed as a microgram per mL of the sample.

#### Enzymatic Antioxidants

##### Ascorbate peroxidase (APX) activity

Ascorbate peroxidase activity was measured by homogenizing seeds in 50 mM potassium phosphate buffer (pH 7). A previously defined method (Dixit et al., 2001) was followed to measure APX activity. The buffer used in this assay was prepared by mixing 200 mM potassium phosphate buffer (pH 7.0), 10 mM ascorbic acid, and 0.5 M ethylenediamine tetraacetic acid (EDTA). This buffer was then mixed with H_2_O_2_ (1 mL) and supernatant 50 μl. the APX activity was recorded by recording the decrease in absorbance at 290 nm every 30 s (Chen and Asada, 1989).

##### Catalase (CAT) activity

Catalase activity was estimated by homogenizing seed samples in medium composing 50 mM potassium phosphate buffer (pH 7.0) and 1 mM dithiothreitol (DTT) Catalase (CAT) (Beers and Sizer, 1952). The assay solution was prepared by mixing 50 mM phosphate buffer (pH 7.0), 59 mM H_2_O_2_, and 0.1 mL enzyme extract. The decrease in absorbance of the reaction solution at 240 nm was recorded after every 20 s. An absorbance change of 0.01 min^–1^ was defined as 1 U of CAT activity. Enzyme activity was selected on the basis of seed weight.

##### Peroxidase (POD) activity

For Peroxidase determination, seeds were homogenized in a medium consisting of 50 mM potassium phosphate buffer (pH 7.0), 0.1 M EDTA, and 1 mM DTT. Peroxidase activity was measured following a previously described method (Hameed et al., 2014). The assay solution contained distilled water (545 μl), 200 mM phosphate buffer (pH 7.0), 200 mM guaiacol, 400 mM H_2_O_2_, and 15 μl enzyme extract. The reaction was initiated by adding enzyme extract. At 470 nm, the increase in absorbance of reaction was recorded after every 20 s. One unit of POD activity was defined as an absorbance change of 0.01min^–^1. Enzyme activity was expressed on a seed weight basis.

##### Superoxide dismutase (SOD) activity

Superoxide dismutase activity was measured by homogenizing seed samples in a buffer consisting of 50 mM potassium phosphate buffer (pH 7.0), 0.1 mM EDTA, and 1 mM dithiothreitol (DTT) as previously described (Dixit et al., 2001). The SOD activity was measured by its ability to inhibit photochemical reduction of nitro blue tetrazolium (NBT), a protocol followed as previously described (Giannopolitis and Ries, 1977). One unit of SOD activity was defined as the number of enzymes that caused 50% inhibition of the photochemical reduction of NBT.

#### Hydrolytic Enzymes

##### Esterase activity

A previously described method (Van Asperen, 1962) was followed to determine α-esterases and β-esterases activity. Substrates, α- naphthyl acetate, and β-naphthyl acetate was used. The reaction mixture contained a substrate solution [30 MMA orb-naphthyl acetate, 0.04 M phosphate buffer (pH 7) and 1% acetone,] and enzyme extract. In darkness, the mixture was incubated for exactly 15 min at 27°C and then 1 mL of staining solution (1% Fast blue BB and 5% SDS mixed in a ratio of 2:5) was added and incubated in darkness for 20 min at 27°C. The amount of α -and β-naphthol produced was measured by recording the absorbance at 590 nm. Using the standard curve, enzyme activity was an orb naphthol produced in μM min^–^1 per g seed weight.

##### Protease activity

Seed samples were extracted in 50 mM potassium phosphate buffer pH 7.8, for the assessment of protease activity. A casein digestion assay, previously described (Van Asperen, 1962), was used for Protease activity estimation. In this method, one unit is the size of an enzyme, which releases acid-soluble fragments equivalent to 0.001 A280 min^–1^ at 37°C and pH 7.8. Enzyme activity was expressed on a seed weight basis.

##### Alpha-amylase activity

For the estimation of seed alpha-amylase activity, a previously described method (Khalid and Hameed, 2017) was followed with some minor modifications.

#### Other Biochemical Parameters

##### Total oxidant status (TOS)

Total oxidant status (TOS) was determined using a formulated method (Erel, 2005) which is based on the oxidation of ferrous ion to ferric ion by oxidants present in the sample in an acidic medium, and the measurement of ferric ion by xylenol orange (Harma et al., 2005). The assay mixture contained sample extract, reagent R1, and reagent R2. Absorption was measured at 560 nm after 5 min with the help of a spectrophotometer. Hydrogen peroxide was used to draw a standard curve. The results were expressed in μM H_2_O_2_ equivalent per L.

##### Malondialdehyde (MDA) content

In the seed flour level of the lipid, peroxidation was determined in terms of malondialdehyde (MDA, a product of lipid peroxidation) content, determined by the thiobarbituric acid (TBA) reaction following a previously described method (Harma et al., 2005) with minor modifications (Dhindsa et al., 1981). In a 0.1% TCA seed sample, 0.25 g was homogenized. The homogenate was centrifuged at 14,462 × g for 5 min. In the 1 m aliquot of the supernatant 20% TCA containing 0.05%, TBA was added. At 95°C the mixture was heated for 30 min and then quickly cooled in an ice-bath. After centrifuging at 14,462 × g for 10 min, the absorbance of the supernatant at 532 nm was measured and the value for the non-specific absorption at 600 nm was subtracted. The extinction coefficient of 155 mM^–1^ cm^–1^ was used to determine the MDA content.

##### Total antioxidant capacity (TAC)

A previously described method was followed (Ahmed et al., 2020) with minor modifications for the determination of TAC. The ABTS assay represents a decrease of 2,2-azino-bis (3 ethylbenzothiazoline-6- sulfonate) radical cation ABTS+ (blue-green in color) into the original ABTS (colorless compound), due to the presence of antioxidants in the sample. The antioxidants of the sample extract according to their content and decolorize the ABTS+ radical cation. The reaction mixture contains reagent sample extract, R1, and reagent R2. The absorption of each reaction mixture was measured after 5 min at a wavelength of 660 nm. To develop a calibration curve for this analysis, AsA (ascorbic acid) was used. The results for antioxidant contents found in plant extracts were measured as μM AsA equivalent to1 g.

##### Proline content measurement

Proline was determined by following a previously described method (Bates et al., 1973) with minor modifications. Briefly, the ground seed sample was weighed (0.2 g). After adding 2 mL of 3% sulfosalicylic acid in a 10 mL centrifuge tube, it was boiled for 10 min, and the supernatant was used for the proline extract. One milliliter supernatant was reacted with 1 mL glacial acid and 1 mL acid ninhydrin, boiled for 1 h at 100°C, and then the reaction was terminated in an ice bath. The reaction mixture was extracted with a 2 mL toluene, mixed vigorously, and left at room temperature for 30 min until the separation of the two phases. The chromospheres-containing toluene (1 mL, upper phase) was warmed to room temperature and its optical density was measured at 520 nm using toluene as a blank. The proline concentration was determined from a standard curve using D-Proline.

##### Pigment analysis

The concentration of lycopene and carotenoids were determined by a previously described method (Lichtenthaler and Wellburn, 1983). Ground samples (0.2 g) were added in acetone (80%) and centrifuged at 10,000 g for 5 min. Absorbance was measured at 645, 663, and 480 nm using a spectrophotometer.

##### In vitro anti-diabetic activity (α-amylase enzyme inhibition method)

The α-amylase inhibition was determined as per the 3,5-dinitrosalicylic acid (DNSA) method (Miller, 1959). The ground seed samples were dissolved in the lowest amount of 10% DMSO and were then dissolved in 0.02 M Na_2_SO_4_ buffer and 6 mM sodium chloride at pH 6.9 to obtain two final concentrations of 100 μg/mL. The reaction solution comprised 200 μL α-amylase solution and 200 μL of the plant material which was further incubated at 30°C for 10 min. After that, 200 μL of 1% starch solution in distilled H2O (w/v) was mixed with all extracts and incubated for 3 min. 200 μL DNSA reagent (12 g of sodium potassium tartrate tetrahydrate in 8 mL of 2M NaOH and 20 mL of 0.096 M of 3, 5 DNSA solution) was further added to bring the reaction to the end and was then boiled at 90°C in a water bath for 10 min. All the reaction solutions were diluted with 5 mL distilled H2O and the absorbance was measured at 540 nm with a UV-visible spectrophotometer. The blank (control) of 100% enzyme activity was prepared using 200 μL of the buffer instead of the plant extract. Acarbose was used as a standard drug and the reaction was executed similar to that mentioned above with concentrations of μg/mL. The α-amylase inhibition was measured as % inhibition using the following equation:

$$\%\,\mathrm{age}\,\mathrm{inhibition}\,\mathrm{of}\,\alpha -\text{amylase}=\frac{\overset{\text{Enzyme activity of control}}{-\mathrm{Enzyme}\,\mathrm{activity}\,\mathrm{of}\,\mathrm{extract}\times 100}}{\mathrm{Enzyme}\,\mathrm{activity}\,\mathrm{of}\,\mathrm{control}}$$

### In vitro Anti-inflammatory Activity (Protein Denaturation Method)

The anti-inflammation was determined following the inhibition method of albumin denaturation. Previously described methods (Mizushima and Kobayashi, 1968; Sakat et al., 2010) were used to test this activity with minor modifications. An aqueous solution of 1% bovine albumin serum was prepared and then its pH was adjusted to 6.0 with 1M HCL. The reaction solution was a mixture of test extracts with a concentration of 5 mg/mL DMSO to prepare the stock solutions. Diclofenac sodium (10 mg), as a standard drug, was also used to prepare the stock solutions as test samples. These stock solutions of test extracts and standard drugs were further used for the final concentrations of μg/mL. The standard and all the extract solutions were incubated for 20 min at 37°C and then heated at 70°C in a water bath for 5 min. The turbidity was measured at 660 nm with a spectrophotometer after cooling the reaction mixtures. The readings were taken in triplicate. The percentage inhibition of protein denaturation was calculated using the following formula:

$$\%\mathrm{age}\mathrm{inhibition}=\frac{\left[\mathrm{Abs}\,\mathrm{control}-\mathrm{Abs}\,\mathrm{sample}\right]\,\times 100}{\mathrm{Abs}\,\mathrm{control}}$$

### Statistical Analysis

Statistical analysis was performed using XL-STAT software version 2014.1.02 (Copyright Addinsoft 1995-2012)^1^. To organize and analyze the resulting data, descriptive statistics were applied. Data was subjected to analysis of variance (ANOVA) with three replications. Tukey (HSD) test at *P* < 0.05 and analysis of variance was used to test the significance of the data. In the graphs, values presented are mean ± SE. Mean data was subjected to the perform principal component analysis using the same software.

### Results

#### Non-enzymatic Antioxidants

##### Total phenolic content (TPC)

Based on the observed differences in the studied parameters, desi and kabuli genotypes were grouped into three categories i.e., low, medium, and high (Table 2). In the low category, 16 genotypes were placed with a TPC ranging from 6800 to 9700 (μM/g s. wt.). Among these genotypes, 33% were of desi type and 12% were of kabuli type. The lowest value of TPC (6800 ± 550 μM/g s. wt.) was found in desi type WH-12. Out of all tested genotypes, 57 genotypes were grouped in the intermediate category with a value ranging from 10125 to 18975 (μM/g s. wt.) In this class, 42% of the genotypes were of kabuli type and 71% were of desi type. In the high category, 17 genotypes were grouped with values ranging from 19200 to 34725 (μM/g s. wt.) Among these genotypes, 25% were of kabuli type and 17% were of desi type (Supplementary Figure 1). However, among all the tested genotypes, CM-98 (desi type) showed the highest value (34725 ± 275 μM/g s. wt.) for seed TPC (Table 3).

**TABLE 2:** Scale for categorization of Chickpea genetic resource in high, medium, and low value for different biochemical parameters.

	Parameters	Low	Genotypes	Medium	Genotypes	High	Genotypes
1	TPC (μM/g s. wt.)	6800-9700	WH-12 (Desi)	10125-18975	CH64/11(Kabuli)	19200-34725	CH2/11 (Desi)
			Gocke (Kabuli)		AUG-424 (Desi)		WH-4 (Desi)
			Dashat-98 (Desi)		CH49/09 (Desi)		CH35/10 (Desi)
			CH54/07 (Kabuli)		Karak-2 (Desi)		Paidar-91 (Desi)
			NIAB-CH2016 (Desi)		BKK-2174 (Kabuli)		CH13/011 (Desi)
			Thall-2006 (Desi)		CH24/11 (Desi)		sheenghar-2000 (Desi)
			Noor-91 (Kabuli)		CH28/07 (Desi)		NIFA-95 (Desi)
			CM-2008 (Kabuli)		Punjab-2000 (Desi)		CM877/10 (Kabuli)
			CH98/99 (Kabuli)		ICCV-96029 (Desi)		WH-9 (Desi)
			CM1235/08 (Kabuli)		CH56/09 (Kabuli)		CH61/09 (Kabuli)
2	Acid (Vit C) (μg/g s. wt.)	5-24.750	WH-10 (Desi)	31.00-59.750	CH60/10 (Kabuli)	60.00-69.705	BKK-2174 (Kabuli)
			Noor-2009 (Kabuli)		Tamman-2013 (Kabuli)		AUG-424 (Desi)
			WH-12 (Desi)		CH2/11 (Desi)		CH24/07 (Desi)
			ICCV-96030 (Desi)		WH-13 (Desi)		WH-9 (Desi)
			CH10/11 (Desi)		CM407/13 (Desi)		Bitttle-2016 (Desi)
			Balkasar-2000 (Desi)		CM3457/91 (Desi)		WH-11 (Desi)
			CH19/10 (Desi)		CH28/07 (Desi)		CH64/11 (Kabuli)
			Wanhar-2000 (Desi)		CM3384/00 (Desi)		CM877/10 (Kabuli)
			CM-72 (Desi)		CH104/06 (Desi)		WH-5 (Desi)
			CM1681/08 (Desi)		CH77/08 (Kabuli)		Paidar-91 (Desi)
3	TFC (μg/mL sample)	69.8-98.68	CH35/10 (Desi)	102.4-296.13	CH39/08 (Desi)	317.86-394.98	WH-1 (Desi)
			CH39/11 (Desi)		CM407/13 (Desi)		Tamman-2013 (Kabuli)
			WH-14 (Desi)		Karak-3 (Desi)		Noor-2013 (Kabuli)
			CH19/10 (Desi)		CH13/011 (Desi)		WH-11 (Desi)
			ILWC-247 (Desi)		CH63/11 (Kabuli)		
			CM1681/08 (Desi)		WH-16 (Desi)		
			CM3384/00 (Desi)		CH55/09 (Kabuli)		
			WH-15 (Desi)		CH72/08 (Kabuli)		
			WH-13 (Desi)		Punjab-2000 (Desi)		
			CH40/09 (Desi)		Karak-2 (Desi)		
4	APX (Units/g s. wt.)	460-680	WH-8 (Desi)	700-1280	Punjab-91 (Desi)	1340-1680	CH32/10 (Desi)
			CH16/06 (Desi)		Wanhar-2000 (Desi)		CH77/08 (Kabuli)
			Karak-2 (Desi)		CH35/10 (Desi)		CH60/10 (Kabuli)
			Bitttle-2016 (Desi)		CM-98 (Desi)		BKK-2174 (Kabuli)
			CH104/06 (Desi)		CH24/07 (Desi)		Tamman-2013 (Kabuli)
			Thall-2006 (Desi)		WH-10 (Desi)		
			sheenghar-2000 (Desi)		Karak-1 (Desi)		
					CH74/08 (Kabuli)		
					WH-11 (Desi)		
					WH-7 (Desi)		
5	CAT (Units/g s. wt.)	80-104	CM2984/91 (Desi)	109-793	WH-12 (Desi)	800-893	CH13/011 (Desi)
			Tamman-2013 (Kabuli)		ICCV-96030 (Desi)		CH2/11 (Desi)
			Thall-2006 (Desi)		Karak-1 (Desi)		WH-16 (Desi)
			Noor-2013 (Kabuli)		Bitttle-2016 (Desi)		CH10/11 (Desi)
			WH-4 (Desi)		Noor-2009 (Kabuli)		CH56/09 (Kabuli)
			WH-1 (Desi)		Bhakhar-2011 (Desi)		WH-6 (Desi)
			CM-72 (Desi)		WH-2 (Desi)		CH77/08 (Kabuli)
					Lawaghar (Kabuli)		CM407/13 (Desi)
					CH104/06 (Desi)		CM-2000 (Kabuli)
					NIAB-CH2016 (Desi)		ICC-4951 (Desi)
6	POD (Units/g s. wt.)	649.10 - 999	Noor-2009 (Kabuli)	1015.5-1998.5	CH13/011 (Desi)	2031.3-2564.1	WH-14 (Desi)
			CM3457/91 (Desi)		CH28/07 (Desi)		CH40/09 (Desi)
			ICCV-96029 (Desi)		CM1681/08 (Desi)		CM3384/00 (Desi)
			Lawaghar (Kabuli)		Karak-1 (Desi)		CH49/09 (Desi)
			WH-6 (Desi)		Noor-2013 (Kabuli)		CM1235/08 (Kabuli)
			CM2984/91 (Desi)		ILWC-247 (Desi)		
			CH16/06 (Desi)		CH35/10 (Desi)		
			NIAB-CH2016 (Desi)		Dashat-98 (Desi)		
			WH-13 (Desi)		Noor-91 (Kabuli)		
			Punjab-2008 (Desi)		Parbat-98 (Desi)		
7	SOD (Units/g s. wt.)	24.09-59.92	WH-3 (Desi)	62.08-195.63	sheenghar-2000 (Desi)	201.77-279.76	CH35/10 (Desi)
			Bitttle-98 (Desi)		Thall-2006 (Desi)		CH10/11 (Desi)
			Punjab-2000 (Desi)		CH32/10 (Desi)		CH40/09 (Desi)
			WH-7 (Desi)		Karak-3 (Desi)		CH68/08 (Kabuli)
			Bitttle-2016 (Desi)		NIFA-88 (Desi)		Lawaghar (Kabuli)
			Noor-91 (Kabuli)		WH-12 (Desi)		CM-72 (Desi)
			WH-6 (Desi)		CM-2000 (Kabuli)		Bhakhar-2011 (Desi)
			C-44 (Desi)		WH-11 (Desi)		WH-13 (Desi)
			Karak-1 (Desi)		Paidar-91 (Desi)		Noor-2013 (Kabuli)
			CH24/07 (Desi)		Karak-2 (Desi)		CM407/13 (Desi)
8	Esterase (μM/min/g s. wt.)	17.32-18.98	CH98/99 (Kabuli)	19.03-29.41	CH49/09 (Desi)	30.67-37.05	Noor-2009 (Kabuli)
			ICC-4951 (Desi)		WH-16 (Desi)		CH104/06 (Desi)
			CH2/11 (Desi)		CM-2000 (Kabuli)		Noor-2013 (Kabuli)
			BKK-2174 (Kabuli)		CM1681/08 (Desi)		CH56/09 (Kabuli)
			CH35/10 (Desi)		Parbat-98 (Desi)		
			Lawaghar (Kabuli)		CH68/08 (Kabuli)		
			Dashat-98 (Desi)		CM877/10 (Kabuli)		
			Gocke (Kabuli)		NIFA-95 (Desi)		
			CH10/11 (Desi)		CH55/09 (Kabuli)		
			CM-88 (Desi)		CM3444/92 (Desi)		
9	Protease (Units/g s. wt.)	3400-5557	Tamman-2013 (Kabuli)	6395-10040	Punjab-2000 (Desi)	100910-11080	CH2/11 (Desi)
			CM-72 (Desi)		Parbat-98 (Desi)		Thall-2006 (Desi)
			Wanhar-2000 (Desi)		WH-2 (Desi)		Karak-2 (Desi)
					Noor-91 (Kabuli)		
					CM-2008 (Kabuli)		
					sheenghar-2000 (Desi)		
					Paidar-91 (Desi)		
					Karak-3 (Desi)		
					NIFA-88 (Desi)		
					Dashat-98 (Desi)		
10	Alpha amylase (mg/g s. wt.)	7.35-26.79	CM-2008 (Kabuli)	31.89-155.84	Bitttle-2016 (Desi)	165.09-213.02	CH54/07 (Kabuli)
			CM3444/92 (Desi)		Karak-3 (Desi)		WH-7 (Desi)
			Bitttle-98 (Desi)		CH64/11 (Kabuli)		CH60/10 (Kabuli)
			Parbat-98 (Desi)		CH24/11 (Desi)		sheenghar-2000 (Desi)
			NIFA-95 (Desi)		CM877/10 (Kabuli)		C-44 (Desi)
			CH74/10 (Kabuli)		CH10/11 (Desi)		CH74/08 (Kabuli)
					CM1051/11 (P1) (Desi)		
					CH68/08 (Kabuli)		
					CH24/07 (Desi)		
					CM3457/91 (Desi)		
11	TOS (μM/g s. wt.)	25-95	CM-72 (Desi)	112-296	Paidar-91 (Desi)	300-356	NIAB-CH2016 (Desi)
			WH-5 (Desi)		CH61/09 (Kabuli)		CH104/06 (Desi)
			WH-1 (Desi)		CH32/10 (Desi)		CH74/08 (Kabuli)
			Thall-2006 (Desi)		Karak-3 (Desi)		CM-2008 (Kabuli)
			Gocke (Kabuli)		CM407/13 (Desi)		NIFA-88 (Desi)
			CH40/09 (Desi)		sheenghar-2000 (Desi)		Punjab-2000 (Desi)
			BKK-2174 (Kabuli)		Bitttle-98 (Desi)		CM877/10 (Kabuli)
			ICC-4951 (Desi)		Bhakhar-2011 (Desi)		WH-7 (Desi)
			CH19/10 (Desi)		WH-13 (Desi)		WH-12 (Desi)
					WH-11 (Desi)		CH74/10 (Kabuli)
12	MDA (μM/g f. wt.)	188.9-199.74	NIFA-88 (Desi)	201.29-254.70	WH-7 (Desi)	255.48-295.74	CH39/08 (Desi)
			Punjab-2000 (Desi)		Punjab-2008 (Desi)		CM877/10 (Kabuli)
			WH-3 (Desi)		Gocke (Kabuli)		WH-16 (Desi)
			WH-11 (Desi)		Dashat-98 (Desi)		ILWC-247 (Desi)
			Bitttle-98 (Desi)		CM-72 (Desi)		CH98/99 (Kabuli)
			WH-4 (Desi)		sheenghar-2000 (Desi)		CH61/09 (Kabuli)
			WH-12 (Desi)		Bitttle-2016 (Desi)		WH-15 (Desi)
			WH-5 (Desi)		WH-9 (Desi)		CH55/09 (Kabuli)
			Noor-2009 (Kabuli)		Balkasar-2000 (Desi)		CH2/11 (Desi)
			Karak-2 (Desi)		WH-10 (Desi)		CH72/08 (Kabuli)
13	TAC (μM/g s. wt.)	0.355-2.963	CM3457/91 (Desi)	2.94-6.963	ICCV-96029 (Desi)	7.400-8.35	CH40/09 (Desi)
			CM1051/11 (P1) (Desi)		CH24/07 (Desi)		Gocke (Kabuli)
			CH68/08 (Kabuli)		Karak-1 (Desi)		Thall-2006 (Desi)
			Lawaghar (Kabuli)		CM-2000 (Kabuli)		WH-1 (Desi)
			WH-10 (Desi)		CM3444/92 (Desi)		WH-5 (Desi)
			CH28/07 (Desi)		CH64/11 (Kabuli)		CM-72 (Desi)
			WH-4 (Desi)		WH-2 (Desi)		
			CH35/10 (Desi)		C-44 (Desi)		
			CH13/011 (Desi)		AUG-424 (Desi)		
			CM-88 (Desi)		Balkasar-2000 (Desi)		
14	Proline (μg/g s. wt.)	148.857-156.714	CH32/10 (Desi)	157.71-209	CH104/06 (Desi)	211-272.500	CH19/10 (Desi)
			BKK-2174 (Kabuli)		CM877/10 (Kabuli)		WH-8 (Desi)
			CH39/08 (Desi)		ICCV-96030 (Desi)		CH74/08 (Kabuli)
			CH56/09 (Kabuli)		CH98/99 (Kabuli)		Parbat-98 (Desi)
			CH72/08 (Kabuli)		CH55/09 (Kabuli)		Bitttle-2016 (Desi)
			Gocke (Kabuli)		WH-16 (Desi)		ILWC-247 (Desi)
			CM1681/08 (Desi)		Noor-91 (Kabuli)		WH-3 (Desi)
					ICCV-96029 (Desi)		CH24/11 (Desi)
					Bitttle-98 (Desi)		WH-12 (Desi)
					CM-2000 (Kabuli)		WH-5 (Desi)
15	lycopene (μg/g s. wt.)	1.159-1.989	CH24/11 (Desi)	2.018-9.620	CM3444/92 (Desi)	10.557-12.579	CH32/10 (Desi)
			CM877/10 (Kabuli)		Bitttle-2016 (Desi)		CH55/09 (Kabuli)
			CH98/99 (Kabuli)		CH77/08 (Kabuli)		Karak-3 (Desi)
			CM-2008 (Kabuli)		WH-15 (Desi)		sheenghar-2000 (Desi)
			CH74/10 (Kabuli)		CH2/11 (Desi)		
			AUG-424 (Desi)		WH-16 (Desi)		
			Lawaghar (Kabuli)		WH-7 (Desi)		
			CH64/11 (Kabuli)		WH-5 (Desi)		
			ILWC-247 (Desi)		ICCV-96029 (Desi)		
			CH28/07 (Desi)		WH-4 (Desi)		
16	Total carotenoids (μg/g s. wt.)	31.42-32.70	CH98/99 (Kabuli)	33.21-49.75	CH54/07 (Kabuli)	51.03-58.43	CH61/09 (Kabuli)
			CH74/10 (Kabuli)		CH24/11 (Desi)		Punjab-2000 (Desi)
			CM877/10 (Kabuli)		CH28/07 (Desi)		Karak-3 (Desi)
			CM-2008 (Kabuli)		CH2/11 (Desi)		CH32/10 (Desi)
					ILWC-247 (Desi)		CH55/09 (Kabuli)
					CH68/08 (Kabuli)		sheenghar-2000 (Desi)
					AUG-424 (Desi)		
					WH-15 (Desi)		
					WH-5 (Desi)		
					CH64/11 (Kabuli)		
17	Anti-diabetic activity (% inh)	64.90-69.99	CH49/09 (Desi)	70.63-79.89	CH24/11 (Desi)	80.10-82.33	WH-4 (Desi)
			CH63/11 (Kabuli)		sheenghar-2000 (Desi)		Balkasar-2000 (Desi)
			CH35/10 (Desi)		CH40/09 (Desi)		Wanhar-2000 (Desi)
			Punjab-2000 (Desi)		WH-13 (Desi)		CH74/10 (Kabuli)
			CH68/08 (Kabuli)		CM3444/92 (Desi)		Noor-91 (Kabuli)
			Punjab-91 (Desi)		CH64/11 (Kabuli)		CM-2008 (Kabuli)
			CH98/99 (Kabuli)		CH32/10 (Desi)		NIFA-95 (Desi)
			BKK-2174 (Kabuli)		Bitttle-2016 (Desi)		Thall-2006 (Desi)
			CH10/11 (Desi)		Lawaghar (Kabuli)		CH56/09 (Kabuli)
					CM1681/08 (Desi)		CM-88 (Desi)
18	Anti-inflammatory activity (% inh)	45.51-46.61	Thall-2006 (Desi)	56.35-77.79	CM3457/91 (Desi)	78.55-78.88	Parbat-98 (Desi)
			CM3384/00 (Desi)		CM2984/91 (Desi)		Bhakhar-2011 (Desi)
			CH2/11 (Desi)		CH72/08 (Kabuli)		CM-72 (Desi)
					BKK-2174 (Kabuli)		WH-6 (Desi)
					WH-16 (Desi)		ICCV-96030 (Desi)
					CH74/10 (Kabuli)		WH-1 (Desi)
					CH28/07 (Desi)		
					CH39/08 (Desi)		
					CH19/10 (Desi)		
					Punjab-2008 (Desi)		

**TABLE 3:** Maximum values for different parameter in Chickpea seed.

Sr#	Variable	Maximum value	Genotypes
1	TPC	34725 μM/g s. wt.	CM-98 (Desi)
2	AsA	69.23 μg/g s. wt.	WH-3 (Desi)
3	TFC	394.98 μg/mL sample	WH-11 (Desi)
4	APX	1680 Units/g s. wt.	Tamman-2013 (Kabuli)
5	Catalase	4030 Units/g s. wt.	ICC-4951 (Desi)
6	POD	2564.10 Units/g s. wt	CM1235/08 (Kabuli)
7	SOD	279.76 Units/g s. wt	CH24/11 (Desi)
8	Esterase	37.05 μM/min/g s. wt.	CH56/09 (Kabuli)
9	protease	11080 μM/min/g s. wt.	Karak-2 (Desi)
10	Alpha amylase	213.01 mg/g s. wt.	CH74/08 (Kabuli)
11	TOS	356.5 μM/g s. wt.	CM3457/91 (Desi)
12	MDA	295.74 μM/g s. wt.	CM-2008 (Kabuli)
13	TAC	8.35 μM/g s. wt	CM-72 (Desi)
14	Proline	272.50 μg/g s. wt.	ICC-4951 (Desi)
15	lycopene	12.57 μg/g s. wt	sheenghar-2000 (Desi)
16	Total carotenoids	58.43 μg/g s. wt.	sheenghar-2000 (Desi)
17	Anti- diabetic Activity	82.00%	CM-88 (Desi)
18	Anti-Inflammatory Activity	78.88%	WH-1 (Desi), WH-6 (Desi), ICCV-96030 (Desi)

*TPC, total phenolic content; AsA, ascorbic acid; TFC, total flavonoid content; APX, ascorbate peroxidase; CAT, catalase; POD, peroxidase; SOD, superoxide dismutase; TOS, total oxidant status; MDA, malondialdehyde; TAC, total antioxidant capacity.*

##### Ascorbic acid (AsA)

A significant variation in seed ascorbic acid (AsA) content provided the base for the categorization of tested genotypes in low, medium, and high groups (Table 2). In the high category, there were 16 genotypes with values ranging from 60 to 69.23 μg/g s. wt. Among these genotypes, 14% were of desi type and 29% were of kabuli type (Supplementary Figure 2). The highest seed AsA content (69.23 ± 2.25 μg/g s. wt.) was observed in a desi type i.e., WH-3 (Table 3). In the intermediate category, 60 genotypes with AsA values ranging from 31.0 to 59.75 (μg/g s. wt.) were grouped. Among these, 63% of genotypes were of kabuli type and 68% were of desi type. In the low category 14 genotypes were found, 27% of which had values ranging from 5.00 to 24.75 (μg/g s. wt.). In the low category, 18% desi type and 8%kabuli type genotypes were grouped while the lowest ascorbic acid value (5.0 ± 1.0 μg/g s. wt.) was detected in a desi type genotype i.e., WH-10.

##### Total flavonoid content (TFC)

A significant variation was observed among genotypes for seed total flavonoid content (TFC) (Supplementary Figure 3). In the low category, 15 genotypes were placed with TFC ranging from 68.80 to 98.69 μg/mL samples. Among these genotypes, 18% were of desi type and 13% were of kabuli type. Desi type CH35/10 showed the lowest value of TFC (69.80 ± 6.61 μg/mL sample). In the intermediate category, 71 genotypes were grouped, with a value ranging from 102.40 to 296.13 μg/mL sample. In this class, 79% of the genotypes belonged to the kabuli type and 79% were of desi type. In the high category, four genotypes were grouped, with values ranging from 317.86 to 394.98 μg/mL sample. Among these genotypes, 8% were of kabuli type and 3% were of desi type. Among all tested genotypes, WH-11 (desi type) showed the highest value (394.98 ± 13.06 μg/mL sample) for seed TFC (Table 3).

#### Enzymatic Antioxidants

##### Ascorbate peroxidase (APX) activity

A significant variation in seed ascorbate peroxidase (APX) activity was provided as the base for the categorization of tested genotypes in low, medium, and high groups (Table 2). In the high category there were five genotypes with values ranging from 1340 to 1680 Units/g s. wt. (Supplementary Figure 4). Among these genotypes, 2% were of desi type and 17% were of kabuli type. The highest seed ascorbate peroxidase Activity (1680 ± 40 Units/g s. wt.) was observed in a kabuli type chickpea genotype i.e., Tamman-2013 (Table 3). In the medium class, 78 genotypes with an APX value ranging from 700 to 1280 (Units/g s. wt) were grouped. Among these, 83% of the genotypes were of kabuli type and 88% were of desi type. In the low category, there were seven genotypes with values ranging from 460 to 680 Units/g s. wt. All genotypes grouped in the low category were of desi type and they made up 10% of the total desi genotypes under investigation, while the lowest APX activity (460 ± 60 Units/g s. wt.) was detected in genotype i.e., WH-8.

##### Catalase (CAT) activity

Based on observed differences in studied parameters, desi and kabuli genotypes were grouped into three categories i.e., low, medium, and high (Table 2). In the low category, seven genotypes were placed with CAT ranging from 80 to 104 Units/g s. wt. Among these genotypes, 8% were of desi type and 8% were of kabuli type. The lowest CAT activity (80 ± 7 Units/g s. wt.) was detected in desi type CM2984/91. Intermediate CAT activity was found in 69 genotypes with a value ranging from 109 to 793 Units/g s. wt. In this class, 67% of the genotypes belonged to the kabuli type and 80% were of desi type. High CAT activity was detected in 14 genotype values ranging from 800 to 893 Units/g s. wt. Among these genotypes, 25% were of kabuli type and 12% were of desi type (Supplementary Figure 5). Among all tested genotypes, ICC-4951 (desi type) showed the highest value (893 ± 50 Units/g s. wt.) for seed CAT activity (Table 3).

##### Peroxidase (POD) activity

A significant variation in seed peroxidase (POD) activity was provided as the base for the categorization of tested genotypes in low, medium, and high groups (Table 2). The data shows (Supplementary Figure 6) that in the high category there were five genotypes with values ranging from 2031.30 to 2564.10 Units/g s. wt. Among these genotypes, 6% were of desi type and 4% were of kabuli type. Overall, the highest seed peroxidase (POD) activity (2564.10 ± 233.10 Units/g s. wt.) was observed in a kabuli type chickpea genotype i.e., CM1235/08 (Table 3). Intermediate POD activity was found in 72 genotypes with POD values ranging from 1015.50 to 1998.50 (Units/g s. wt.). Among these, 83% of the genotypes were of kabuli type and 79% were of desi type. In the low category, there were 13 genotypes with values ranging from 649.10 to 24.75 (unit/g s. wt). Low POD activity was detected in 13% kabuli and 15% desi chickpea genotypes and on the whole, the lowest peroxidase (POD) activity (649.10 ± 16.90 unit/g s. wt.) was detected in kabuli type genotype i.e., Noor-2009.

##### Superoxide dismutase (SOD) activity

Based on the significant differences observed in studied parameter, desi and kabuli genotypes were grouped into three categories i.e., low, medium, and high (Table 2). For seed superoxide dismutase (SOD) activity in the low category, 10 genotypes were placed with SOD ranging from 24.10 to 59.92 (Units/g s. wt.). Among these genotypes, 14% were of desi type and 4% were of kabuli type. The lowest value of SOD (24.10 ± 2.10 Units/g s. wt.) was found in desi type WH-3 (Supplementary Figure 2). Intermediate SOD activity was detected in 48 genotypes with a value ranging from 62.08 to 195.64 Units/g s. wt. In this class, 50% of the genotypes belonged to the kabuli type and 55% were of desi type. In the high category 32 genotypes were grouped with values ranging from 201.77 to 279.76 Units/g s. wt. Among these genotypes, 46% were of kabuli type and 31% were of desi type. Among all tested genotypes, CH24/11 (desi type) showed the highest value (279.76 ± 50 Units/g s. wt.) for seed SOD activity (Table 3).

#### Hydrolytic Enzymes

##### Esterase activity

A significant variation in seed esterase activity was provided as the base for the categorization of tested genotypes in low, medium, and high groups (Table 2). The data shows (Supplementary Figure 8) that in the high category there were four genotypes with values ranging from 30.67 to 37.055 μM/min/g s. wt. Among these genotypes, 2% were of desi type and 13% of kabuli type. In general, the highest esterase activity (37.05 μM/min/g s. wt.) was found in kabuli type CH56/09 (Table3). In the medium class, 69 genotypes with esterase values ranging from 19.031 to 29.41 (μM/min/g s. wt.) were grouped. Intermediate esterase activity was found in 58% kabuli type and 85% desi chickpea genotypes. In the low category, there were 17 genotypes with values ranging from 17.32 to 18.98 (μM/min/g s. wt.). Low esterase activity was detected in 13% desi and 29% kabuli chickpea, while the lowest activity (17.32 ± 0.545 μM/min/g s. wt.) was detected in kabuli type genotype i.e., CH98/99.

##### Protease activity

A significant variation was observed among genotypes for seed protease activity (Supplementary Figure 9). Three genotypes were placed in the low category with protease activity ranging from 3400 to 5575 Units/g s. wt. Among these genotypes, 3% were of desi type and 4% of kabuli type. Kabuli type Tamman-2013 showed the lowest value of Protease Activity (3400 ± 250 Units/g s. wt.). In the intermediate category, 84 genotypes were grouped with a value ranging from 6395 to 1040 Units/g s. wt. In this class, 96% of genotypes belonged to the kabuli type and 92% were of desi type. In the high category, three genotypes were grouped with values ranging from 10910 to 11080 Units/g s. wt, and all were of desi type and made up 5% of the total desi genotypes. Among all tested genotypes, Karak-2 (desi type) showed the highest value (11080 ± 10 Units/g s. wt.) for seed protease activity (Table 3).

##### Alpha-amylase activity

A significant variation in seed alpha-amylase activity was provided as the base for the categorization of tested genotypes in low, medium, and high groups (Table 2). The data shows (Supplementary Figure 10) that in the high category there were six genotypes with values ranging from 165.09 to 213.02 mg/g. s. wt. Among these genotypes, 6% were of desi type and 8% were of kabuli type. The highest seed α-amylase activity (213.02 ± 3.20 mg/g. s. wt.) was observed in kabuli type chickpea genotype i.e., CH74/08 (Table 3). In the medium class, 78 genotypes with alpha-amylase activity values ranging from 31.88 to 155.84 (mg/g. s. wt.) were grouped. Among these, 79% of the genotypes were of kabuli type and 89% were of desi type. In the low category, there were six genotypes found with values ranging from 7.35 to 31.88 (mg/g. s. wt.). Low activity was found in 13% kabuli type and 5% desi type, while the lowest activity (7.35 ± 0.566 mg/g. s. wt.) was detected in kabuli type genotype i.e., CM-2008.

#### Other Biochemical Parameters

##### Total oxidant status (TOS)

Based on observed differences in the studied parameters, desi and kabuli genotypes were grouped into three categories i.e., low, medium, and high (Table 2). For seed total oxidant status (TOS) in the low category, nine genotypes were placed with a TOS ranging from 25 to 95 μM/g. s. wt. Among these genotypes, 11% were of desi type and 8% were of kabuli type. Overall, the lowest TOS value (25 ± 0.50 μM/g. s. wt.) was detected in desi type CM-72 (Supplementary Figure 11). In the intermediate category, 59 genotypes were grouped with values ranging from 112 to 296 μM/g. s. wt. In this class, 63% of the genotypes belonged to the kabuli type and 67% were of desi type. In the high category, 22 genotypes were grouped with values ranging from 300 to 356 μM/g. s. wt. Among these genotypes, 29% were of kabuli type and 22% were of desi type. Overall, the highest TOS (356 ± 17.50 μM/g. s. wt.) was detected in desi type CM3457/91 (Table 3).

##### Malondialdehyde (MDA) content

A significant variation in seed malondialdehyde (MDA) content provided the base for the categorization of tested genotypes in low, medium, and high groups (Table 2). The data shows (Supplementary Figure 12) that in the high category, there were 21 genotypes with values ranging from 255.48 to 295.74 μM/g s. wt. Among these genotypes, 18% were of desi type and 38% were of kabuli type. Overall, the highest seed MDA content (295.74 ± 3.097 μM/g s. wt.) was observed in a kabuli type chickpea genotype i.e., CM-2008 (Table 3). In the medium class, 57 genotypes with MDA values ranging from 201.29 to 254.71 (μM/g s. wt.) were grouped. Among these, 58% of genotypes were of kabuli type and 65% were of desi type. In the low category, there were 12 genotypes with values ranging from 188.90to 199.74 (μM/g s. wt.). Low MDA was found in 4% of kabuli types and 17% of desi types. Overall, the lowest MDA content (188.90 ± 1.55 μM/g s. wt.) was detected in desi type genotype i.e., NIFA-88.

##### Total antioxidant capacity (TAC)

Based on observed differences in the studied parameters, desi and kabuli genotypes were grouped into three categories i.e., low, medium, and high (Table 2). In the low category, 22 genotypes were placed with TAC ranging from 0.355 to 2.153** μ**M/g s. wt. Among these genotypes, 22% were of desi type and 29% were of kabuli type. Lowest TAC (0.355 ± 0.044** μ**M/g s. wt.) was observed in desi type i.e., CM3457/91 (desi). In the intermediate category, 62 genotypes were grouped with values ranging from 2.936 to 6.93 μM/g s. wt. In this class, 67% of the genotypes belonged to the kabuli type and 70% were of the desi type. In the high category, six genotypes were grouped with values ranging from 7.40 to 8.36** μ**M/g s. wt. Among these genotypes, 4% were of kabuli type and 8% were desi type (Supplementary Figure 13). Among all tested genotypes, CM-72 (desi type) showed the highest value (8.36 ± 0.082** μ**M/g s. wt.) for seed TAC (Table 3).

##### Proline content

A significant variation in seed proline content provided the base for the categorization of tested genotypes in low, medium, and high groups (Table 2). The data shows (Supplementary Figure 14) that in the high category, there were 12 genotypes with values ranging from 211 to 272.50 (μg/g s. wt.). Among these genotypes, 16% were of desi type and 4% were of kabuli type. On the whole, the highest seed proline content (272.50 ± 20.82 μg/g s. wt.) was observed in a desi type of the chickpea genotype i.e., ICC-4951(Table 3). In the medium class, 71 genotypes with proline content values ranging from 157.71 to 209 (μg/g s. wt.) were grouped. Among them, 79% genotypes were of kabuli type and 79% were of desi type. In the low category, there were seven genotypes with values ranging from 148.85 to 156.71 (μg/g s. wt.). Low proline content was found in 17% kabuli and 5% desi. Overall, the lowest (148.85 ± 8.71 μg/g s. wt.) proline content was detected in a desi genotype i.e., CH32/10.

#### Pigment Analysis

##### Lycopene content

A significant variation was observed between genotypes for seed lycopene content (Supplementary Figure 15). In the low category, 16 genotypes were placed with lycopene values ranging from 1.156 to 1.989 μg/g s. wt. Among these genotypes, 12% were of desi type and 33% were of kabuli type. The lowest value of lycopene (1.159 ± 0.172 μg/g s. wt.) was shown in desi type CH24/11. In the intermediate category, 70 genotypes were grouped with values ranging from 2.018 to 9.620 μg/g s. wt. Intermediate lycopene content was detected in 63% of kabuli and 83% of desi types. In the high category, four genotypes were grouped with values ranging from 10.557 to 12.579 μg/g s. wt. High lycopene content was observed in 4% of kabuli and 5% of desi type genotypes. Overall, the highest lycopene content (12.579 ± 0.313 μg/g s. wt.) was found in desi type Sheenghar-2000 (Table 3).

##### Total carotenoids

A significant variation in seed total carotenoid content provided the base for the categorization of tested genotypes in low, medium, and high groups (Table 2). The data shows (Supplementary Figure 16) that in the high category, there were six genotypes with values ranging from 51.033 to 58.430 μg/g s. wt. Among these genotypes, 6% were of desi type and 8% were of kabuli type. Overall, the highest seed total carotenoid content (58.430.23 ± 0.569 μg/g s. wt.) was observed in a desi type of chickpea genotype i.e., sheenghar-2000 (Table 3). In the intermediate category, 80 genotypes with carotenoids values ranging from 33.216 to 49.75 (μg/g s. wt.) were grouped. Among these, 75% genotypes were of kabuli type and 94% were of desi type. In the low category, there were four genotypes with values ranging from 31.42 to 32.70 (μg/g s. wt.). All the genotypes in the low category were of kabuli type and these made up 17% of the total kabuli genotypes under investigation. However, the lowest carotenoid content (31.42 ± 1.42 μg/g s. wt.) was detected in kabuli type genotype i.e., CH98/99.

#### Therapeutic Analysis

##### *In vitro* anti-diabetic activity (α-amylase inhibition)

A significant variation in seed anti-diabetic activity provided the base for the categorization of tested genotypes in low, medium, and high groups (Table 2). It was found that in the high category there were 10 genotypes with α-amylase inhibition values ranging from 80.10 to 82.33 (%). Among these genotypes, 9% were of desi type and 17% were of kabuli type. The highest seed α-amylase inhibition (82.33 ± 8.06%) was observed in a desi type of chickpea genotype i.e., CM-88, while standard drug acarbose showed 69.35% inhibition (Table 3). In the medium class, 71 genotypes with α-amylase inhibition values ranging from 70.63 to 79.8 (%) were grouped. Among them, 66% of the genotypes were of kabuli type and 83% were of desi type. In the low category, there were nine genotypes with values ranging from 64.90 to 69.99 (%). Among these, 8% desi type and 17% kabuli type genotypes were grouped. The lowest α-amylase inhibition (64.90 ± 3.18%) was detected in desi type genotype i.e., CH49/09 (Supplementary Figure 17).

##### *In vitro* anti-inflammatory activity/albumin inhibition

Based on observed differences in the studied parameter, desi and kabuli genotypes were grouped into three categories i.e., low, medium, and high (Table 2). Three genotypes were placed in the low category with albumin inhibition values ranging from 45.51 to 46.60%, all were of desi type, and they made up 5% of the total desi genotypes under study. Desi type Thall-2006 showed the lowest albumin inhibition at 45.51 ± 1.75% (Supplementary Figure 18). In the intermediate category, 81 genotypes were grouped with values ranging from 56.34 to 77.79%. In this class, 100% of the genotypes belonged to the kabuli type and 86% were of desi type. In the high category, six genotypes were grouped, and values ranged from 78.55to 78.88%, and all were of desi type. Among all tested genotypes, desi type WH-1, WH-6, and ICCV-96030 showed the highest value (78.88 ± 0.55%) for seed albumin inhibition, while standard drug diclofenac sodium showed 81.79% albumin inhibition (Table 3).

### Principal Component Analysis

**FIGURE 1:**
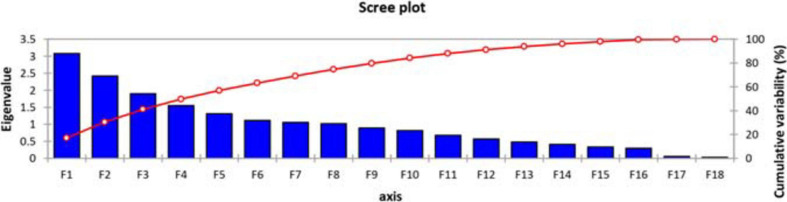
Scree plot representing cumulative variability and Eigenvalues for studied parameters.

For the sake of dimensional data reduction, the transformation of the raw data into principal factors, to analyze the variability among the genotypes, and to obtain the information about inter-relationship among variables, a principal component analysis (PCA) was performed using all parameters under investigation. Scree plot (Figure 1) showed that, among the 18 principal components, eight PC-I, PC-II, PC-III, PC-IV, PC-V, PC-VI, and PC-VII had extracted Eigenvalues > 1. The remaining principal components had Eigenvalues < 1 so have not been discussed further. Cumulatively, these eight principal components contributed 74.67% of the total variation in the genetic resource. Out of a 100% cumulative variability, PC-I and PC-II were the largest contributors with a value of 30.52%, while PC-I, PC-II, PC-III, and PC-1V covered almost 50% of the cumulative variability. Maximum variation was explained by PC-I (17.082%), which was the most important component (Supplementary Table 2).

**FIGURE 2:**
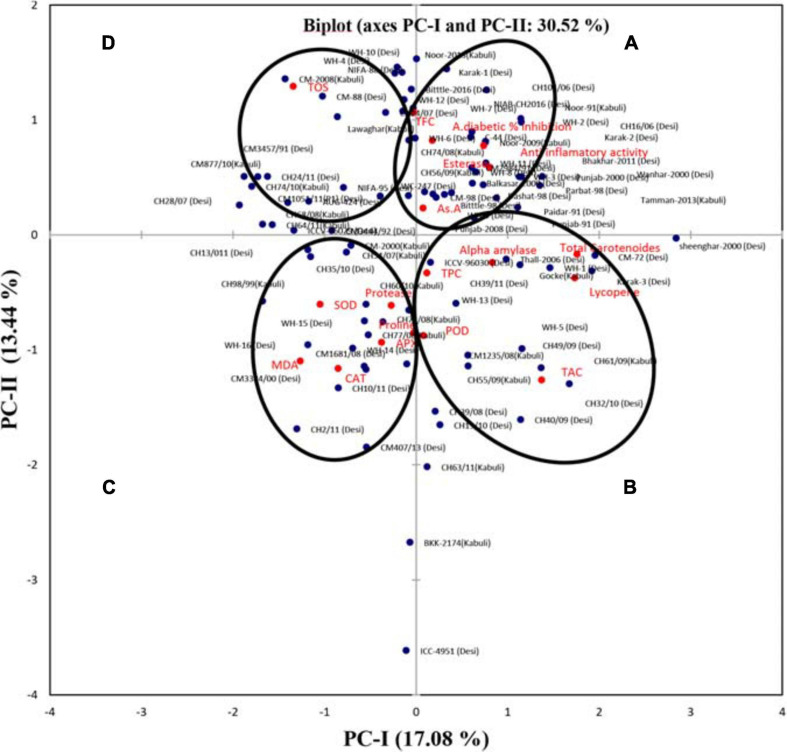
Bi-plot of chickpea genotypes for the first two principal components. Based on the angle between the traits, the biplot was categorized into four groups (**A, B, C**, and **D**).

A genotype by trait biplot was constructed by plotting the PC-I scores (x-axis) against PC-II scores (y-axis) for each trait and all genotypes (Figure 2). This genotype by trait biplot effectively revealed the visual comparison among all genotypes based on multiple traits, and also showed inter-relationships among the traits. The angles between the vectors and the distance of the genotypes from the origin of the biplot were used to extract important information. If the angle between two trait vectors is < 90° then the correlation between the traits is positive, if the angle is > 90° then traits show a negative correlation, while if the angle is equivalent to 90° then traits show no dependency on each other (Shah et al., 2020). Based on the angle between the vectors, the biplot was categorized into four groups (A, B, C, and D). Group A showed a positive correlation among AsA, esterase, T.FC, anti-inflammatory activity, and alpha-amylase inhibition; group B depicted a positive correlation among lycopene, total carotenoids, alpha-amylase, TPC, and TAC; group C indicated a positive correlation among CAT, POD, SOD, MDA, APX, Protease, and proline traits; while in group D no positive association with other variables was detected for TOS.

## Discussion

In recent years, the assessment of antioxidant potential in leguminous seeds has been of great concern. Studies have shown that the chickpea is a member of the leguminous family and possesses a broad range of polyphenolic compounds (Xing and White, 1996; Sarma et al., 2002; Singh et al., 2003). In Pakistan, among rabi pulses, chickpea is the most grown legume crop, cultivated in all areas of Pakistan, especially desert areas−which contribute to bulk production (Abbas et al., 2017).

To analyze the comprehensive antioxidant and therapeutic potential of the chickpea seed, different biochemical analyses (enzymatic antioxidants, non-enzymatic antioxidants, hydrolytic antioxidants etc.) were performed on the seed flour.

In living organisms excessive production of reactive oxygen species (ROS) in the cell, tissue, and extracellular matrix causes a cascade of reactions to boost the endogenous defense mechanism (enzymatic and non-enzymatic antioxidants, etc.) for the successful inactivation of harmful ROS (Birben et al., 2012; Fernández-Mejía, 2013). Among non-enzymatic antioxidants, total phenolic contents (TPC) and total flavonoids contents (TFC) are the main bioactive compounds; they perform various structural functions in the body and are directly associated with antioxidant activity. They may be present in all parts of plants and are commonly consumed (Asif, 2015; de Camargo et al., 2019). Out of 33 released varieties, 31 revealed intermediate total flavonoid contents (TFC), and two showed high TFC. In general, the highest TFC (394.98 ± 13.06 μg/mL sample) was found in a desi wild hybrid WH-1. Similar results were reported in previous studies, validating that dark chickpea seeds depicted higher TFC than light seeded chickpea seeds, as flavonoids are naturally yellow in color (Segev et al., 2010). While some of the kabuli type genotypes also showed high TFC, contradicting previous findings, it could be due to the darker yellow interior of some kabuli genotypes. In case of TPC, seven varieties revealed low TPC, 21 revealed medium TPC, and five revealed high TPC. The highest TPC (34725 ± 275 μM/g s. wt.) was detected in an approved variety CM-98 (desi type), which was 27% higher than the TPC reported in a wheat variety Bhakkar-2000 (25,383.33 μM/g s.wt.) (Khalid and Hameed, 2017). Previously, the highest TPC was found in chickpea variety Balksar 2000 (1.12 mg GAE/g), while in our study Balkasar 2000 showed low TPC (9525 ± 25 μM/g s. wt.) (Zia-Ul-Haq et al., 2008). On the basis of the present findings, it is suggested that colored chickpeas (desi) could be a potentially efficient food because their antioxidants, TPC, and TFC contents are higher than that found in light-colored seeds (kabuli type). In a previous study, it was shown that an outstanding and positive correlation exists between antioxidant activities and total phenolic contents in desi chickpea seeds (Segev et al., 2010; Rani and Khabiruddin, 2016).

Ascorbic acid (vitamin C) acts as a non-enzymatic antioxidant. It also helps to transport electrons and acts as an antioxidant by neutralizing ROS, restoring the antioxidant form of vitamin E (Beyer, 1994; Prasad and Upadhyay, 2011; McGill and Jaeschke, 2013). Ascorbic acid (AsA) is believed to be one of the most efficient antioxidants against different stresses in plants especially in chickpeas (Zarghamnejad et al., 2014; Sharma et al., 2019; Farooq et al., 2020). Out of 33 released varieties used in this study, five varieties revealed high ascorbic acid, 23 were grouped into the intermediate category, and six varieties showed low AsA. The Highest AsA content (69.23 ± 2.25 μg/g s. wt.) was found in desi wild hybrid WH-3, which was higher than the previously reported AsA in the dry seeds of chickpea 40 μg/1 g (Wallace et al., 2016). It was previously reported that a negligible amount of ascorbic acid was present in chickpea, but during the germination process, AsA increases significantly up to 98.5 μg/1g because of the reactivation of the AsA biosynthesis in seeds (Bains et al., 2014).

In living systems enzymatic antioxidants such as superoxide dismutase (SOD), catalase (CAT), Ascorbate peroxidase (APX), and peroxidase (POD) act as the first line of defense against oxidative stress because they have the strong and quick ability to scavenge free radicals, removing hydroxyl radicals (OH), and detoxifying hydrogen peroxide and oxygen intermediates in the cell (Herbinger et al., 2002; Anwar et al., 2018; Ighodaro and Akinloye, 2018; Kohli et al., 2019). Ascorbate peroxidase (APX) is the most important peroxidase enzyme that helps in H_2_O_2_-scavenging, acting as an electron donor, and protecting cell constituents by eliminating ROS (Gangwar et al., 2014). Low APX activity was detected in four varieties; one showing high- and 33 showing intermediate APX activity. In general the highest activity (1680 ± 40 Units/g s. wt.) was found in the kabuli approved variety Tamman-2013, which was almost 15-fold higher than the previously highest reported APX in a wheat flour of variety PAVON (1,426.67 Units/g s. wt.) (Khalid and Hameed, 2017). In earlier research highest APX (947.5 ± 12.5 Units/g s. wt.) was found in a medicinal plant *T. longipetalus* used for curing kidney diseases (Ahmed et al., 2020), which was lower than APX found in chickpea seed flour. Catalase (CAT) is present in all living organisms, especially in higher plants. It is located in major sites (mitochondria, peroxisomes, chloroplast, and cytosol) of H_2_O_2_ production in the cells exposed to oxidative stress, where it helps catalyze the decomposition of H_2_O_2_ into oxygen and water (Sharma and Ahmad, 2014). Low CAT activity was found in four released varieties, intermediate CAT was found in 28 varieties, and high CAT activity was found only in one released variety. In general, the highest CAT activity (893 ± 50 Units/g s. wt.) was found in a desi exotic line (ICC-4951). Previous research on a medicinal plant known as *Peganum harmala* used in the treatment of some disease e.g. cough, diabetes, depression, and some other human ailments, revealed lower catalase activity (555 units/g) than chickpea seeds (Ahmed et al., 2020). Similar results were reported in a case of wheat seed flour (Khalid and Hameed, 2017). Peroxidases (POD) are enzymes that catalyze an oxidation-reduction reaction, employing free radicals that convert several compounds into the polymerized or oxidized form (McGill and Jaeschke, 2013). Low POD activity was found in six varieties, intermediate activity was found in 27 varieties, while highest POD activity (2564.10 ± 233.10 Units/g s. wt.) was found in a kabuli mutant i.e., CM1235/08. Superoxide dismutases (SOD) are a widely present metallo enzyme in living organisms. It helps in the disproportionation of superoxide anions to generate H_2_O_2_ and O2 and neutralizes oxygen radicals (Yan et al., 2016). Low SOD activity was detected in six varieties, intermediate activity was observed in 17, high SOD activity was detected in 10 released varieties, and the highest SOD activity (279.76 ± 50 Units/g s. wt.) was found in desi advance line CH24/11. POD activity in chickpea seed was lower than in wheat flour (42,579.6 Units/g s. wt.), while similar results were found for SOD activity in wheat flour (Khalid and Hameed, 2017).

In living organisms, hydrolytic enzymes like esterase, protease, and alpha-amylase specifically decompose large molecules into smaller ones through hydrolysis. During this process one H_2_O molecule adds to the substance (Wong et al., 2020). They can also act as a secondary system of antioxidants by repairing DNA and utilizing damaged molecules (Pradedova et al., 2011). Esterases are widely distributed and can be found in a vast variety of living systems, they have considerable power to catalyze the synthesis and hydrolysis of ester bonds from a variety of substrates (Zhong et al., 2020). Low esterase activity was found in six varieties, intermediate activity was detected in 26, and only the approved variety showed high esterase activity. In general, the highest esterase activity (37.055 ± 0.59 μM/min/g s. wt.) was found in kabuli advance line i.e., CH56/09. It plays a major role as an antioxidant. It is used in the baking industry and helps in the synthesis of chiral drugs used for curing certain diseases (Panda and Gowrishankar, 2005). In a medicinal plant *Z. fabago*, esterase activity was (14.3 ± 0.44 μM/min/g s. wt.) reported (Ahmed et al., 2020) which is lower than the esterase activity reported in the present study. The present findings validate that chickpea seeds have higher antioxidant potential. In all stages of a plant life cycle, proteases carry out an imperative function in the overall process of protein turnover (Rani et al., 2011). Low protease activity was found in three released varieties; intermediate and high protease activity was detected in 28 and two approved varieties, respectively. In general, the highest protease activity (11080 ± 10 Units/g s. wt.) was observed in desi-approved variety Karak-2, and similar results were reported in the case of wheat flour (Khalid and Hameed, 2017). Alpha-amylase (E.C.3.2.1.1) is a hydrolase enzyme that catalyzes the hydrolysis of 4-glycosidic linkages, with internal α-1 in starch to succumb products like maltose and glucose. It can be isolated from plants, microorganisms, or animals (Sundarram and Murthy, 2014). Low amylase activity was found in three desi- and one kabuli approved varieties. The intermediate activity was observed in 27 approved varieties, while high activity was observed in two varieties. Maximum activity (213.02 ± 3.20 mg/g s. wt.) was found in a kabuli advance line i.e. CH74/08, which is comparatively lower than wheat flour (292.70 mg/g s. wt.) (Khalid and Hameed, 2017). It was noted that cassava mash waste water is a good source of α-amylase, which is active in a wide range of temperatures and pH (Sundarram and Murthy, 2014).

Generally, the total oxidant status (TOS) of living organisms is employed to estimate the overall oxidation state of the body (Vaiserman et al., 2020). Correspondingly, the total antioxidant status (TAS) is used to determine the overall antioxidant capacity of the body (Sevindik, 2018). Low TOS was detected in two varieties, 24 were revealed as intermediate TOS, and seven approved varieties showed high TOS. The highest TOS (356 ± 17.50 μM/g s. wt.) was detected in desi mutant CM3457/91. Low TAC was detected in six approved varieties, intermediate TAC was found in 25, while high TAC was observed in two approved varieties. The highest TAC (8.36 ± 0.082 μM/gs. wt.) was found in the approved desi variety CM-72. In a previous study, it was reported that both kabuli and desi chickpea have good antioxidant potential but desi genotypes native to Pakistan could be a potentially important legume crop with comparatively high antioxidant potential and low oxidant status (Zia-Ul-Haq et al., 2008). Medicinal plant *F. olivieri* seeds showed a higher TAC value (15.6 ± 2.4 μM/g s. wt.) than the TAC reported in chickpea flour (Ahmed et al., 2020). It has already been proven that the fermentation process with a fungus *Cordyceps militaris* improved the antioxidant capacities of chickpeas and thus us considered to be of great potential for the food industry (Xiao et al., 2014). Moreover, another report showed that stirred bio-yogurt received high values of antioxidant capacity when supplied with chickpea flour (Hussein et al., 2020).

Malondialdehyde (MDA) content used as a lipid peroxidation marker in studies related to oxidative stress, is generally used as an indicator of injury in plant membranes (Morales and Munné-Bosch, 2019). Low MDA content was found in seven approved varieties, 25 showed intermediate MDA, while only one approved variety revealed high MDA. On the whole, the highest MDA (295.74 ± 3.097 μM/g s. wt.) was found in a kabuli type chickpea genotype i.e., CM-2008. High MDA was found more in the kabuli type than the desi type, and this could be due to the high antioxidant activity of the desi type (Zia-Ul-Haq et al., 2008). Instead of damage, MDA can play a positive role in acclimation processes by activating regulatory genes involved in plant defense mechanisms (Tounekti et al., 2011). Proline is used as a protein building block. Moreover, it has also been reported to stabilize and protect reactive oxygen species (ROS) scavenging enzymes and stimulate alternative detoxification pathways in plants (Xing and White, 1996). Intermediate and high proline content was found in 30 and three approved varieties, respectively. In general, the highest seed proline content (272.50 ± 20.82 μg/g s. wt.) was observed in a desi exotic line i.e. ICC-4951, which was higher than the already reported proline content in chickpea (158.9 μg/g) (Bhagyawant et al., 2015).

More than 600 naturally occurring lipophilic pigments are grouped as carotenoids, at least 50 of which occur in plant foods. Two groups of carotenoids are found in human blood. Hydrocarbon carotenoids and lycopene both play a vital role in certain disease treatments, so the discrepancy between the number of carotenoids present in plasma and the diet, demands a selective uptake (Abbo et al., 2005). Low total carotenoid content was found in one released variety, while intermediate and high content was found in three and 29 approved varieties, respectively. In the case of lycopene, low lycopene content was found in six approved varieties, while intermediate and high lycopene was found in 25 and two released varieties, respectively. Overall, the highest total carotenoids (58.43 ± 0.56 μg/g s. wt.) and highest lycopene content (12.579 ± 0.313.μg/g s. wt.) were found in a desi approved variety Sheenghar-2000. It has been reported that chickpea seeds more contained carotenoids than engineered beta-carotene containing “golden rice” (Abbo et al., 2005). In another report, it was revealed that total carotenoid concentration ranged from 44 μg g^–^1 in green cotyledon desi and 22 μg g^–^1 in yellow cotyledon kabuli at 32 days post-anthesis (DPA) (Rezaei et al., 2016), while present research showed about 24% more carotenoids than previous findings.

An alpha-amylase inhibition assay was used to determine the anti-diabetic activity of chickpea seed flour. Comparatively, low inhibition was found in two varieties, while intermediate and high inhibition was detected in 24 and seven approved varieties, respectively. Among all tested genotypes, the highest seed α-amylase inhibition (82.33 ± 8.06%) was observed in a desi-approved variety i.e., CM-88, which was higher than standard drug acarbose (69.35%). It has been proven that chickpea seeds can be used both as a dietary or medicinal supplement because of their favorable hypoglycemic effects. According to a report, isoflavones derived from chickpea reveal favorable hypoglycemic activity (Li et al., 2015). Inflammation is the body’s first reaction to injury or infection and is important for both adaptive and innate immunity. The exploration of natural compounds and phyto constituents that show the ability to interfere with these mechanisms, by preventing an extended inflammation, could be helpful for human health (Milán-Noris et al., 2018). Comparatively low, intermediate, and high albumin inhibition was found in one, three, and 29 approved varieties, respectively. Among all tested genotypes, desi wild hybrid WH-1, WH-6, and ICCV-96030 showed the highest value (78.88 ± 0.55%) for seed albumin inhibition, which behaved just like the standard drug used for comparison. It has been reported that strong anti-inflammatory activity is associated with chickpea seed and seed oil, can be used for ear inflammation, and also helps to prevent bowel inflammatory diseases (Milán-Noris et al., 2018). Out of 18 total studied parameters, desi genotypes i.e., CM-98, WH-3, WH-11, ICC-4951, CH24/11, Karak-2, CM-72, sheenghar-2000, CM-88, WH-1, WH-6, and ICCV-96030 revealed highest values for 13 parameters, while kabuli types Tamman-2013, CM1235/08, CH56/09, and CH74/08 showed highest values for only five remaining parameters. It was inferred that desi genotypes have higher anti-oxidant and therapeutic potential than kabuli type.

A multivariate statistical technique Principal Component Analysis (PCA) is employed to simplify the description of the large data, and to extract the important and useful information from the data set (Rathinavel, 2018). To divide the pattern of variation, PCA was performed on all variables simultaneously. In the present study, out of 18 principal components, eight PCs accounted for 74.67% of the total variation. PC-I, PC-II, PC-III, PC-IV, PC-V, PC-VI, and PC-VII exhibited 17.082, 13.44, 10.56, 8.605, 7.26, 6.18 and 5.64% (Supplementary Table 1) variability, respectively. The most discriminating parameter, with positive vector loading in PC-I was TPC, AsA, lycopene, esterases, POD, alpha-amylase, TAC, total carotenoids, anti-inflammatory activity, and % alpha-amylase inhibition. Depending on individual loading, one variable is usually selected from the recognized parameter (Mishra et al., 2015). Carotenoids showed the highest factor loadings 0.787, followed by lycopene with a 0.776-factor loading value, so, the total carotenoids could be the best individual factor loadings selection. PC-II depicted positive factor loadings for six traits i.e., AsA, TOS, TFC esterase, anti-inflammatory activity, and % alpha-amylase inhibition, while TOS could be the best selection for individual factor loadings with a maximum value 0.577 followed by TFC. Distance between genotype and biplot origin measures genotypic differences from the grand mean, so genotypes with long and short distances from the origin can be used to determine best or poorest performers in the environment (Hagos and Abay, 2013). Results revealed that genotypes i.e., ICC-4951(desi), BKK-2174(kabuli), CH63/11(desi), CM407/13(desi), CH2/11(desi), CH19/10, CH40/09, Sheenghar 2000(desi), CM2008(kabuli), and Noor-2013 were found distant from the biplot origin, showing better performance concerning other genotypes with reference to all traits under investigation. The genotypes found near the origin of the biplot were poorer performers than the distant genotypes.

## Conclusion

We conclude that both desi and kabuli genotypes have prominent antioxidants and therapeutic potential, while desi genotypes are more promising than kabuli genotypes. Identified genotypes with the highest and least values for studied parameters can be utilized in breeding programs to design specific breeding strategies to improve these traits in chickpea. Moreover, genotypes with high antioxidant and therapeutic potential can be directly utilized as a natural source of antioxidants to boost the endogenous immune system, as a medicine for diabetes, and in reducing different types of inflammation in the body.

## Data Availability Statement

The original contributions presented in the study are included in the article/Supplementary Material, further inquiries can be directed to the corresponding author/s.

## Author Contributions

SJ did the overall execution of the experiment, analytical work, collection of data, organization of resulting data, write up, and revision of manuscript. AH performed the basic idea and planning, designed the experiments, helped the data analysis, and wrote, revised, and finalized the manuscript. TS performed the basic idea and planning of the experiments and wrote, revised, and finalized the manuscript. All authors contributed to the article and approved the submitted version.

## Conflict of Interest

The authors declare that the research was conducted in the absence of any commercial or financial relationships that could be construed as a potential conflict of interest.

## Footnotes

1 http://www.xlstat.com
