# Supplementary material for: Biochemical Profiling for Antioxidant and Therapeutic Potential of Pakistani Chickpea (Cicer arietinum L.) Genetic Resource
Source: Front Plant Sci. 2021 Apr 13;12:663623. doi: 10.3389/fpls.2021.663623 (PMC8076736; doi:10.3389/fpls.2021.663623)
Supplement: Supplementary file 1 [file Data_Sheet_1.pdf]

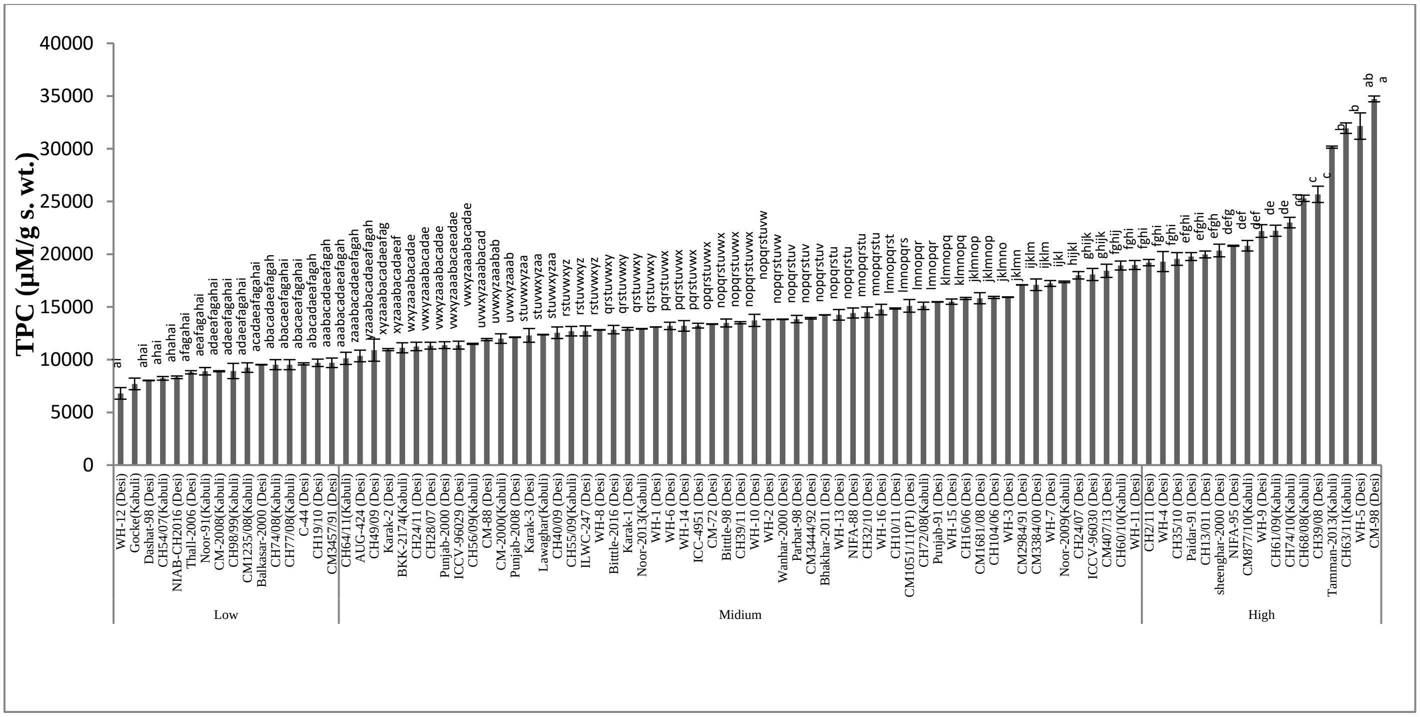

Figure S1: Comparison of seed total phenolic contents (TPC) in chickpea genotypes (mean value ± SD). Means with different alphabets are significantly different (Tukey’s HSD, p<0.05).

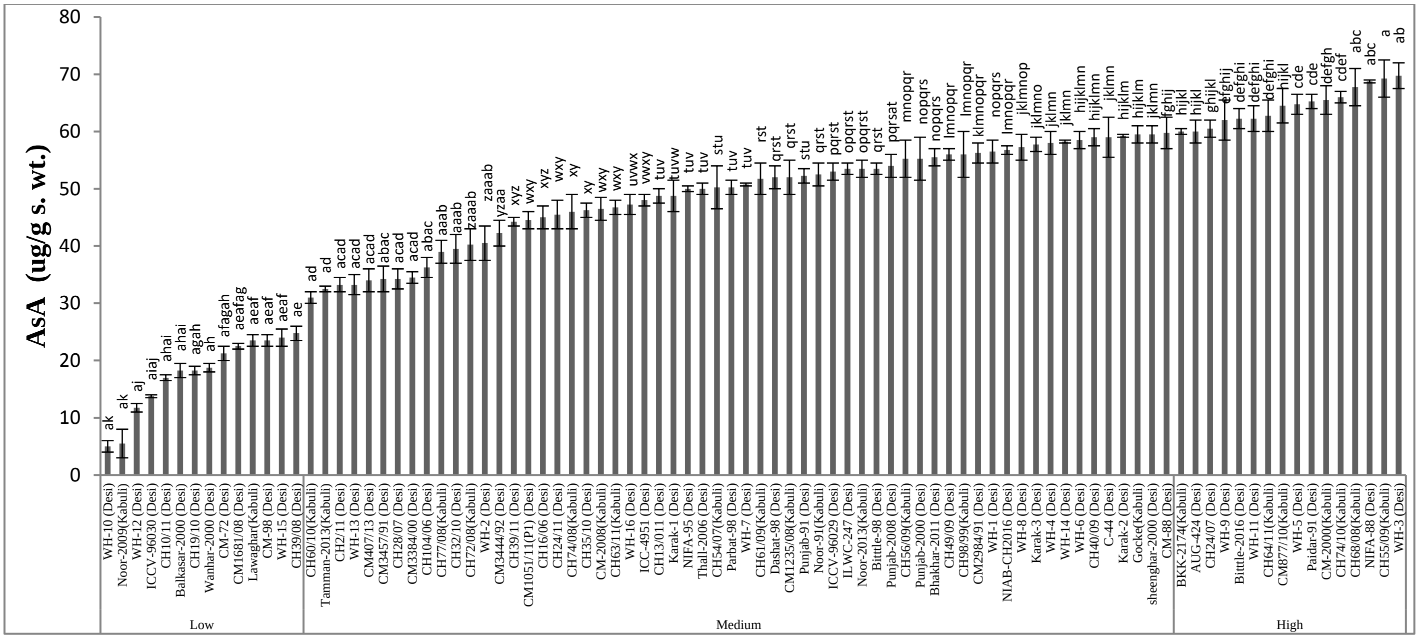

Figure S2: Comparison of seed ascorbic acid (As.A) in chickpea genotypes (mean value ± SD). Means with different alphabets are significantly different (Tukey’s HSD, p<0.05).

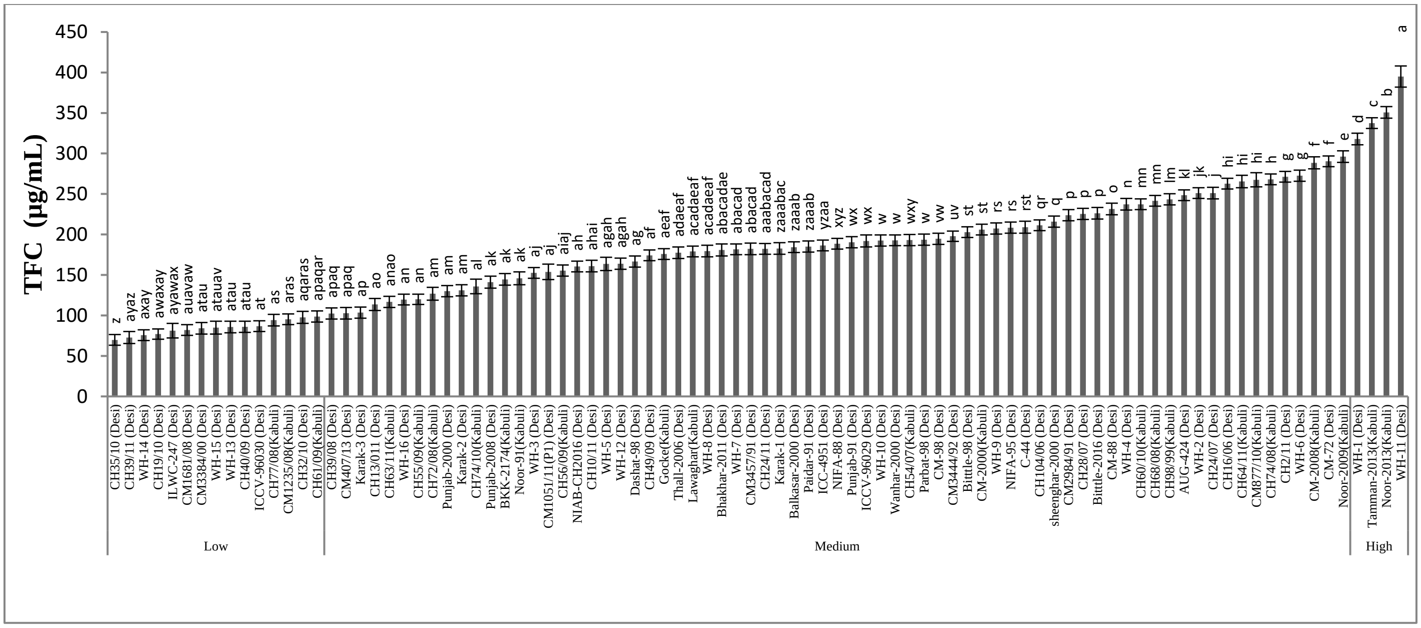

Figure S3: Comparison of seed TFC in chickpea genotypes (mean value ± SD). Means with different alphabets are significantly different (Tukey's HSD, p<0.05).

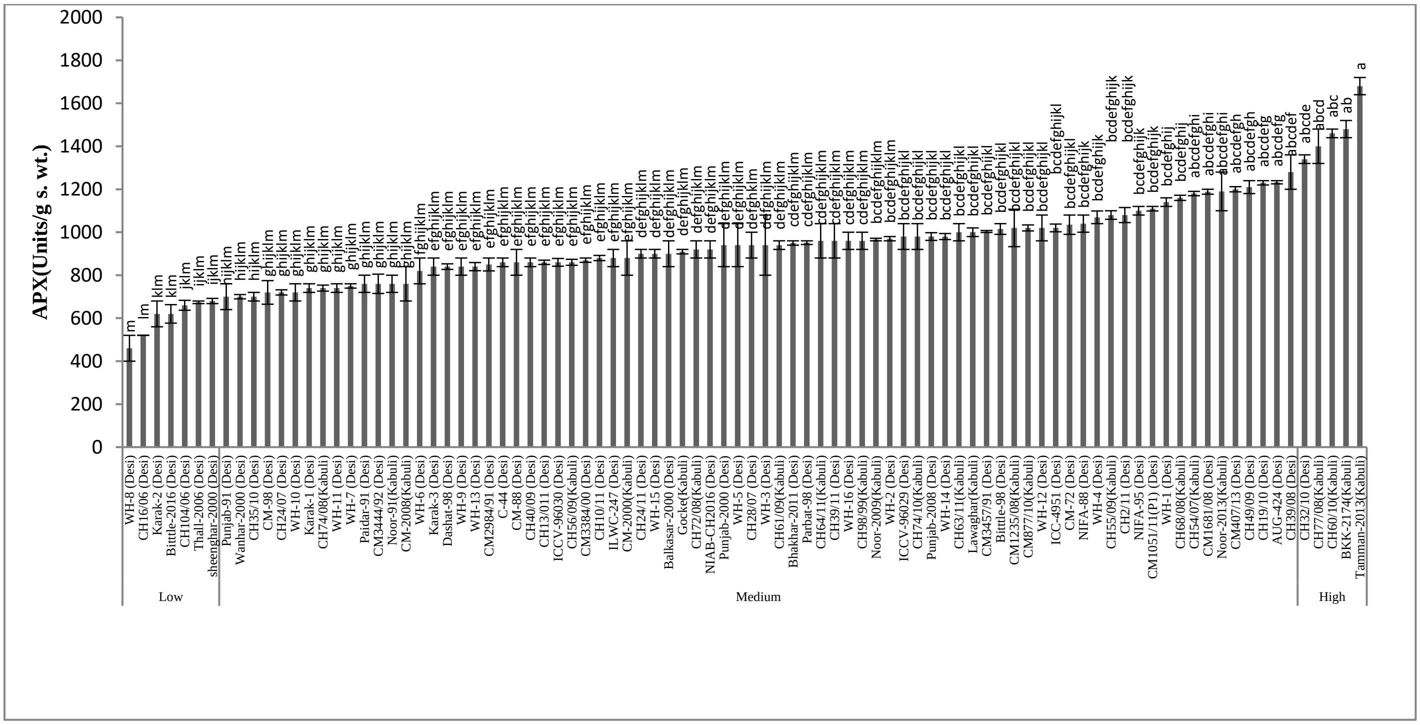

Figure S4: Comparison of seed ascobate peroxidase (APX) in chickpea genotypes (mean value ± SD). Means with different alphabets are significantly different (Tukey's HSD, p<0.05).

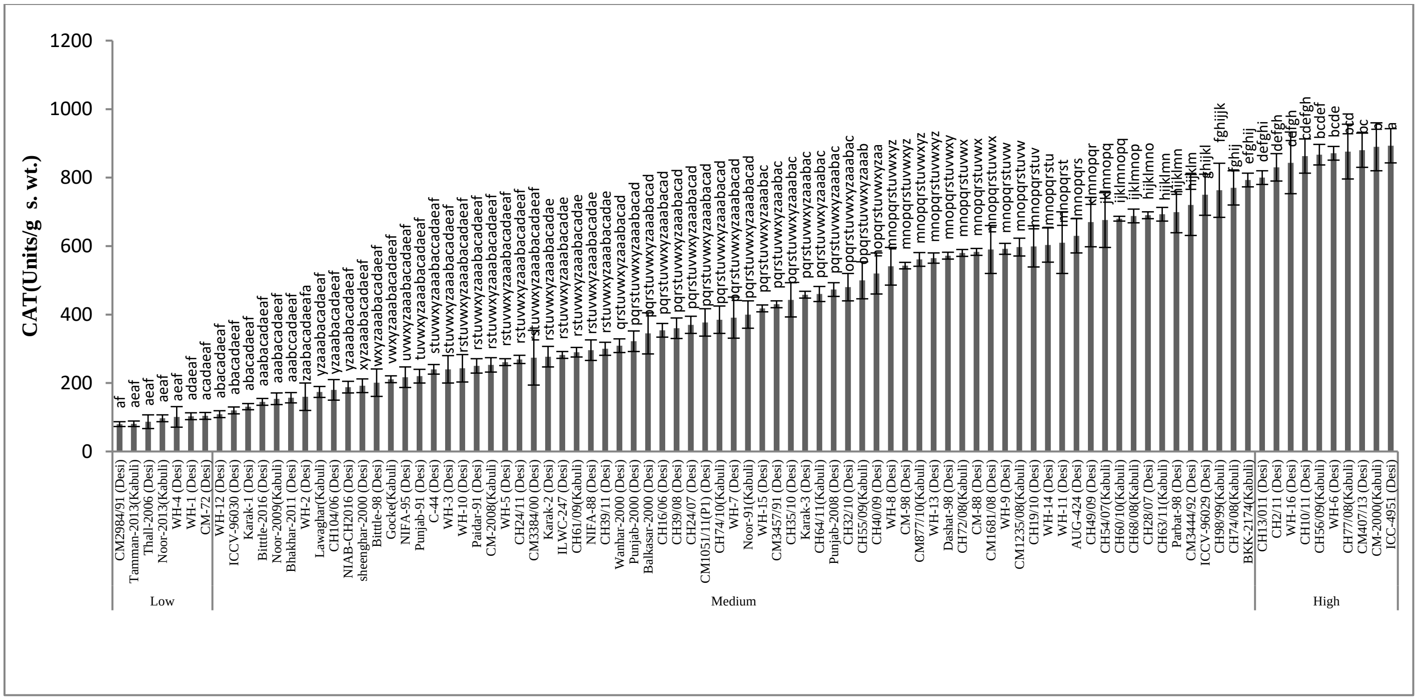

Figure S5: Comparison of seed catalase (CAT) in chickpea genotypes (mean value ± SD). Means with different alphabets are significantly different (Tukey’s HSD, p<0.05).

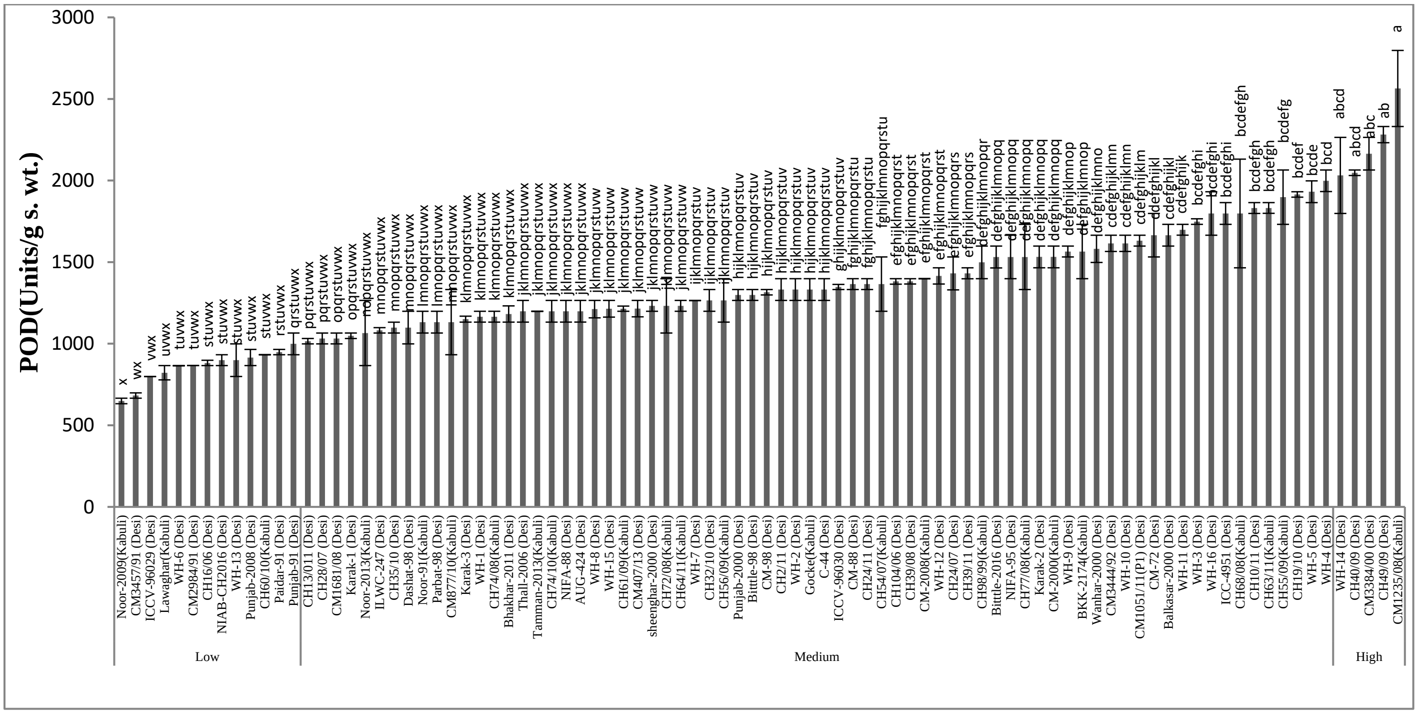

Figure S6: Comparison of seed Peroxidase (POD) activity in chickpea genotypes (mean value ± SD). Means with different alphabets are significantly different (Tukey’s HSD, p<0.05).

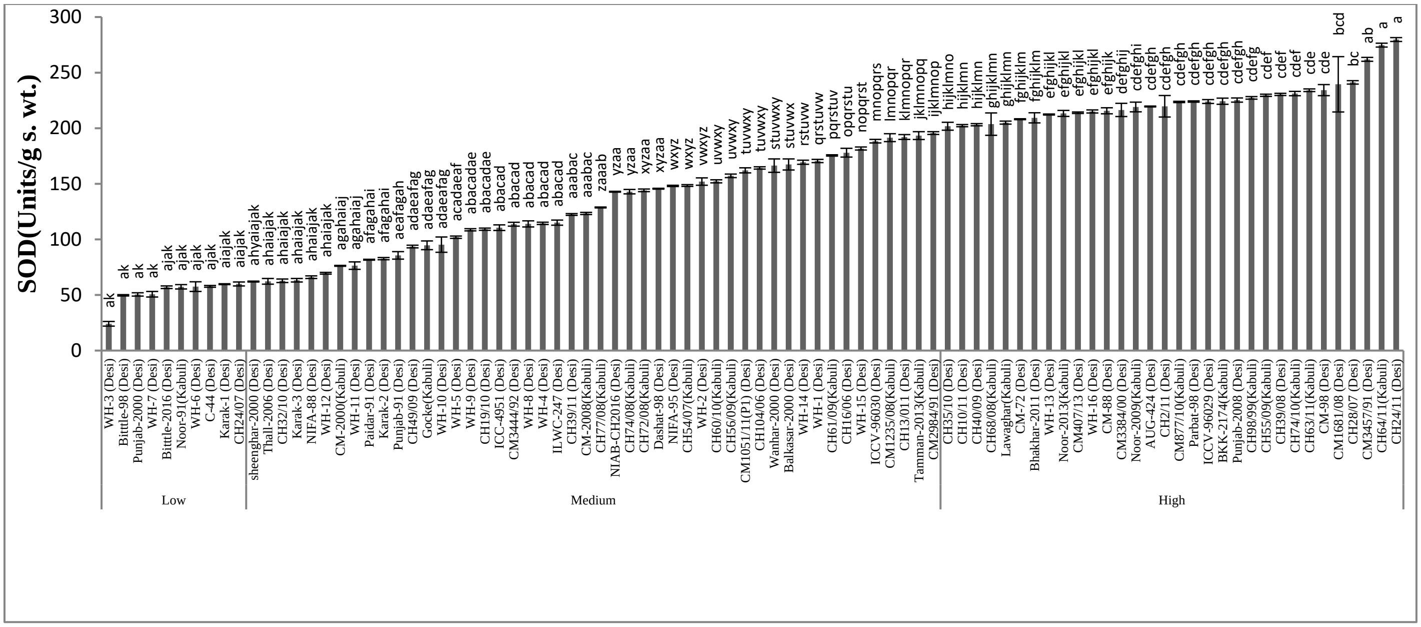

Figure S7: Comparison of seed Superoxide dismutase (SOD) in chickpea genotypes (mean value ± SD). Means with different alphabets are significantly different (Tukey's HSD, p<0.05).

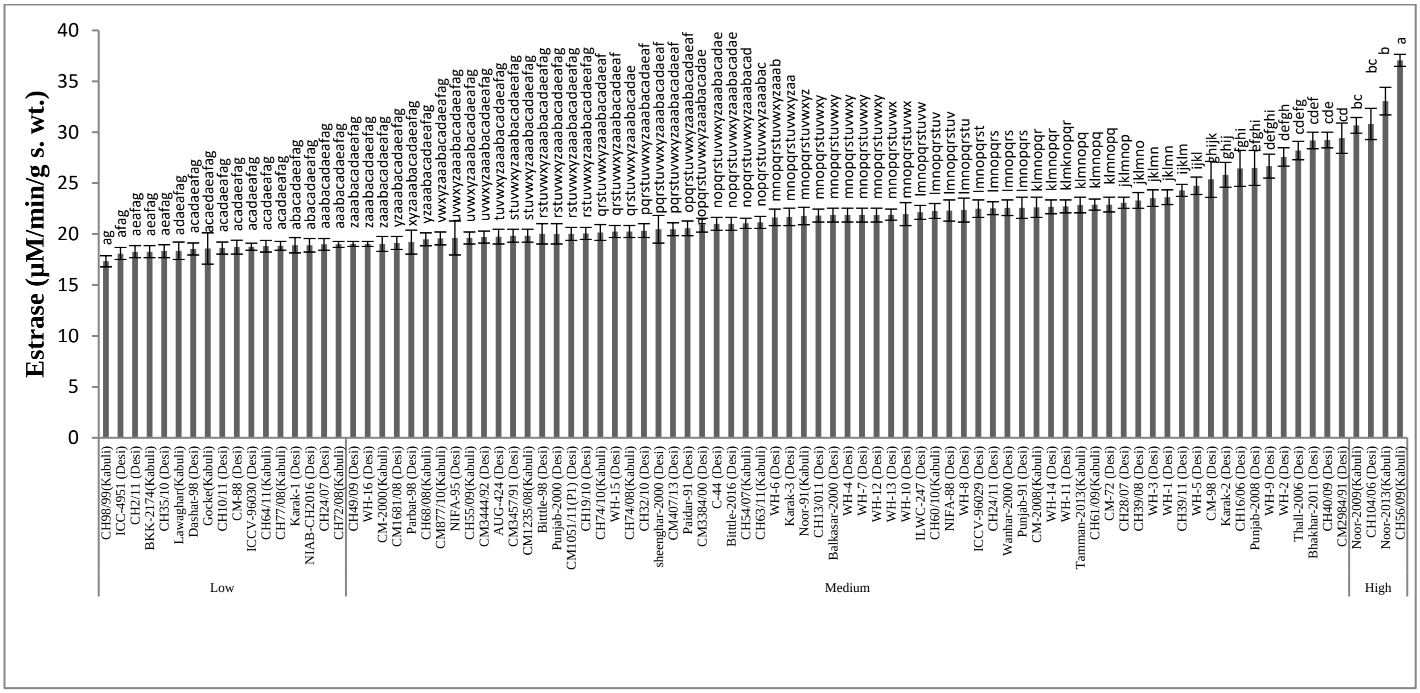

Figure S 8: Comparison of seed esterase activity in chickpea genotypes (mean value ± SD). Means with different alphabets are significantly different (Tukey's HSD, p<0.05).

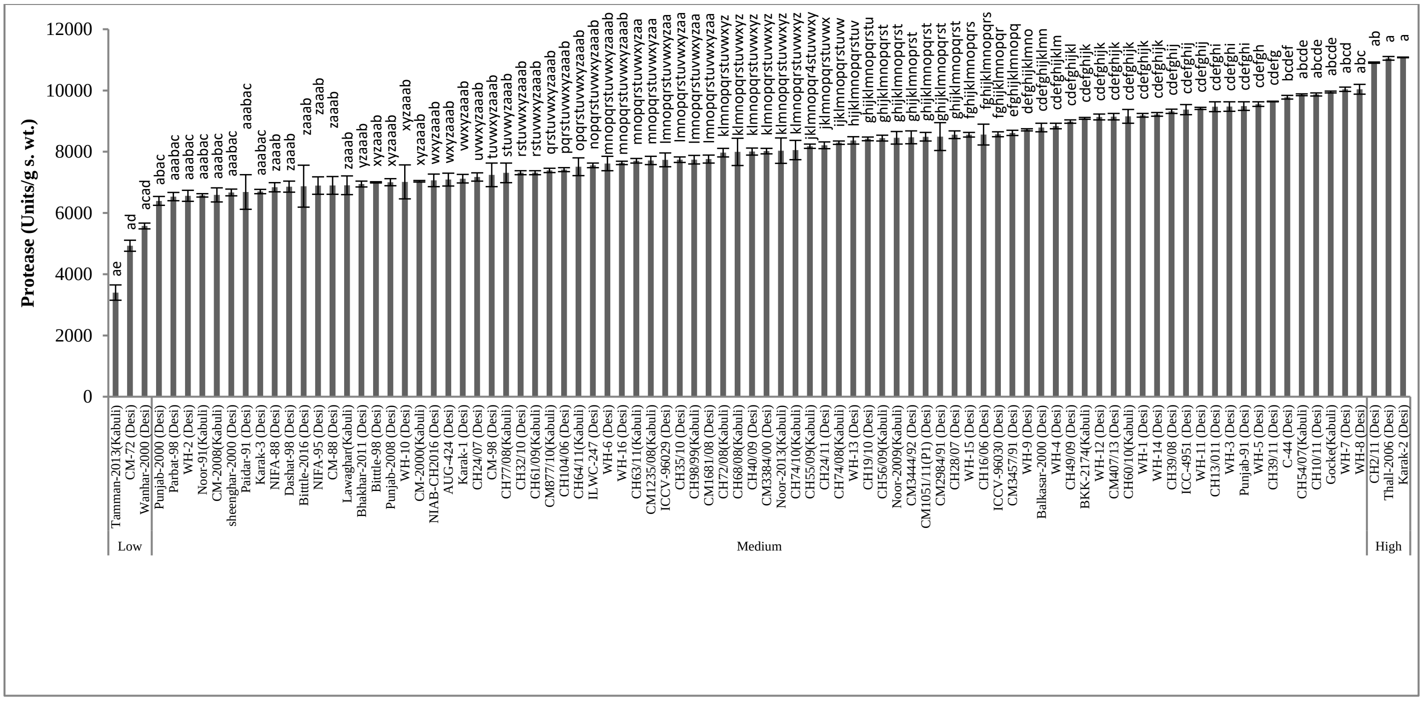

Figure S9: Comparison of seed protease activity in chickpea genotypes (mean value ± SD). Means with different alphabets are significantly different (Tukey's HSD, $p<0.05$).

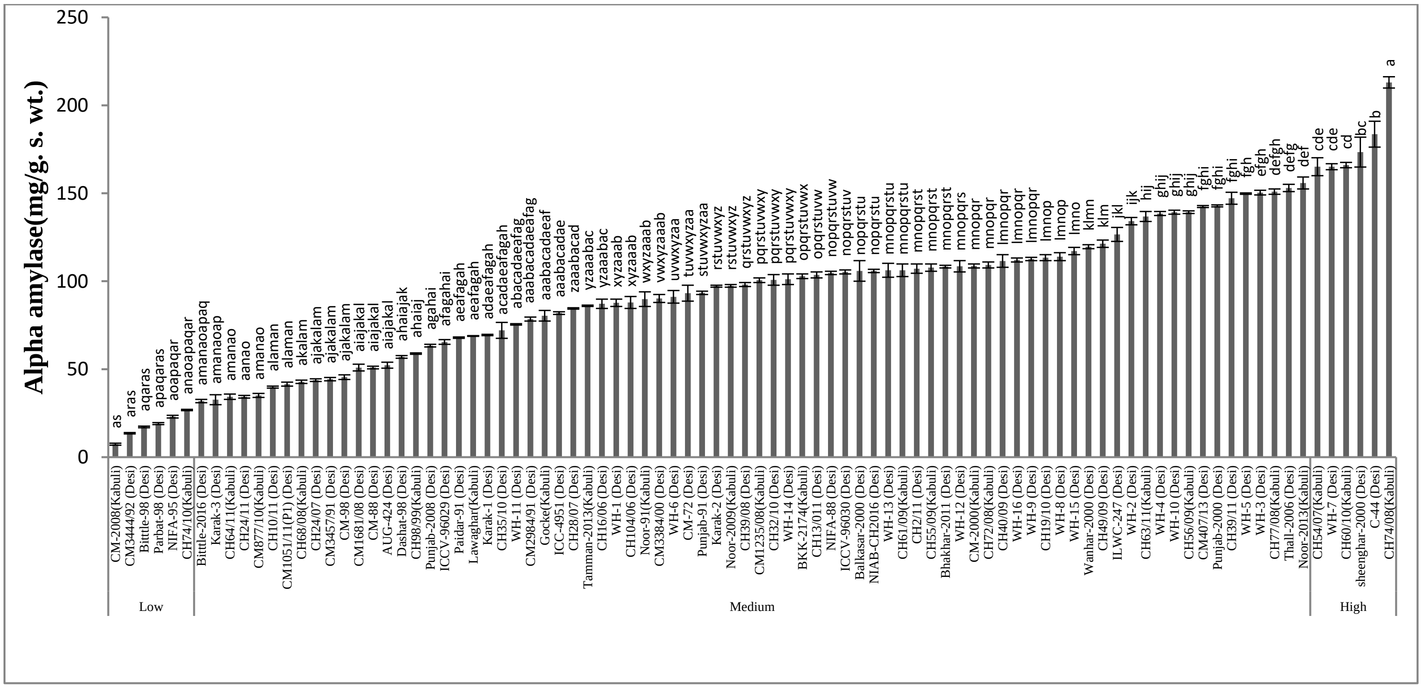

Figure S10: Comparison of seed Alpha amylase in chickpea genotypes (mean value ± SD). Means with different alphabets are significantly different (Tukey's HSD, $p<0.05$).

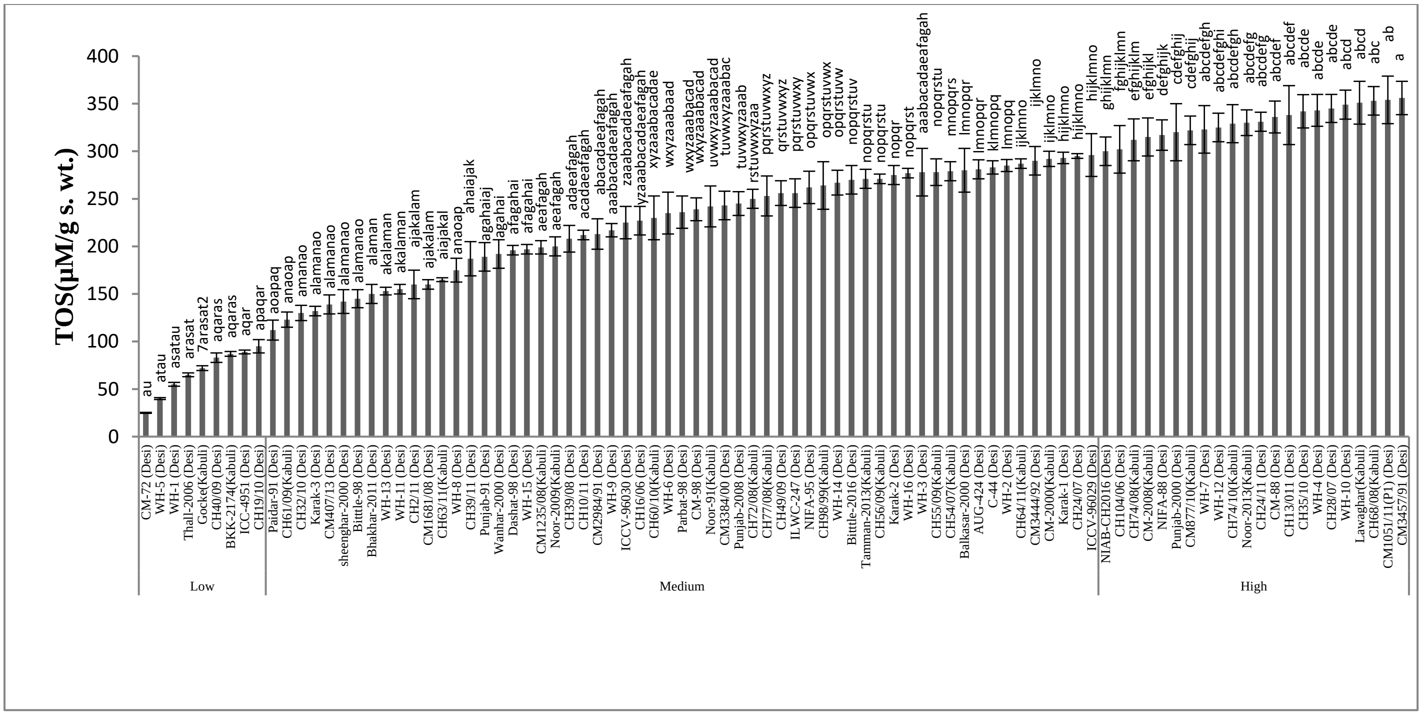

Figure S11: Comparison of seed total oxidant status (TOS) in chickpea genotypes (mean value ± SD). Means with different alphabets are significantly different (Tukey’s HSD, p<0.05).

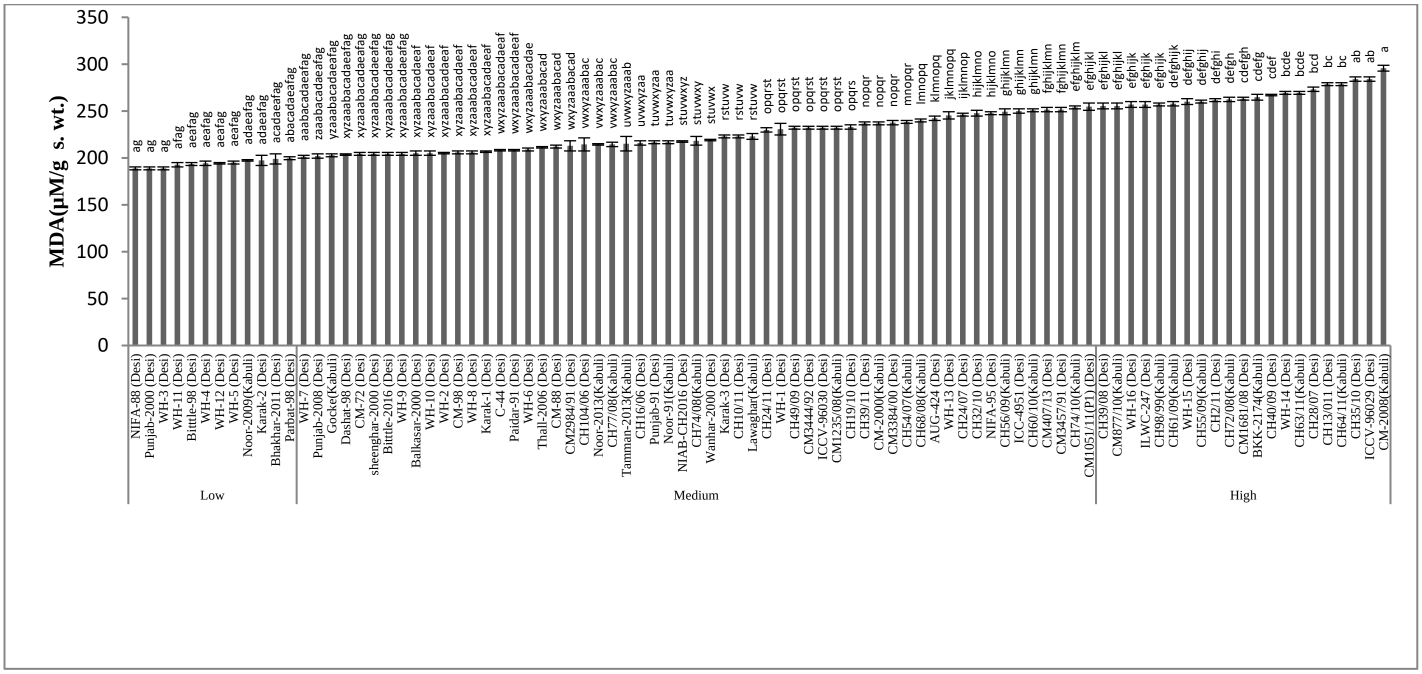

Figure S12: Comparison of seed Malondialdehyde (MDA) Content in chickpea genotypes (mean value ± SD). Means with different alphabets are significantly different (Tukey’s HSD, p<0.05).

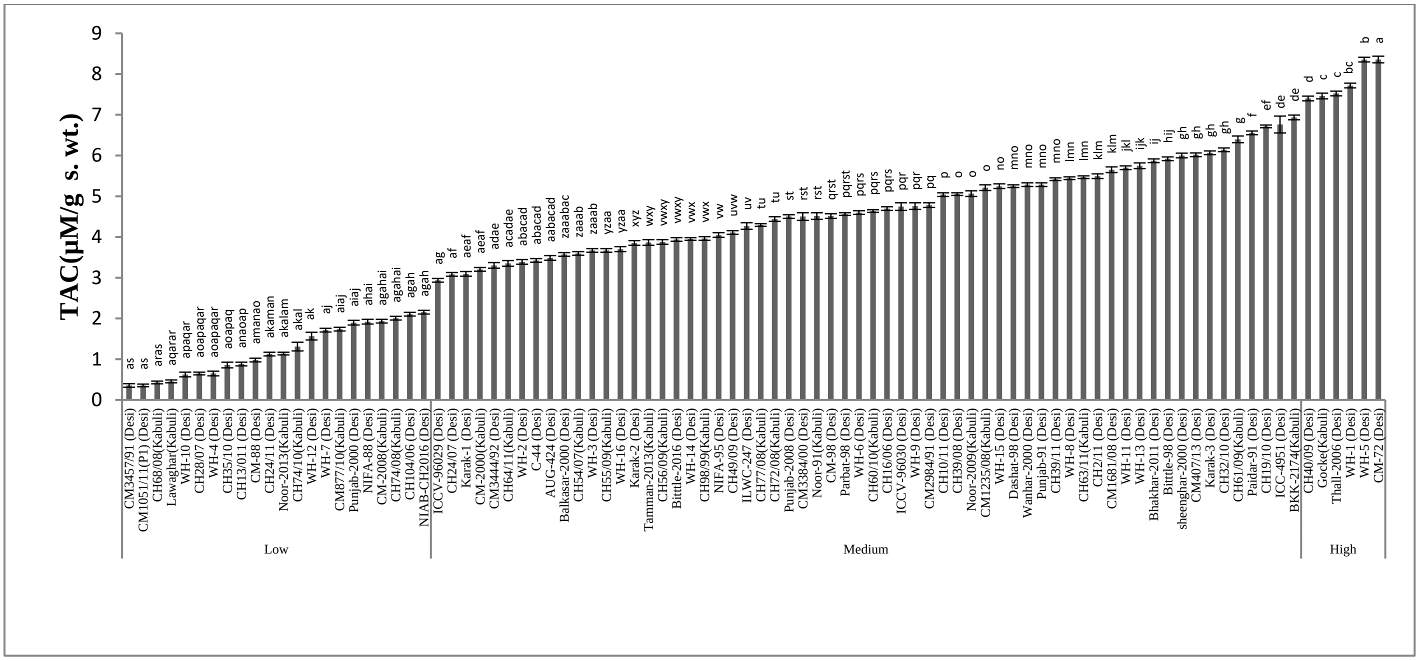

Figure S13: Comparison of seed total antioxidant capacity (TAC) in chickpea genotypes (mean value ± SD). Means with different alphabets are significantly different (Tukey's HSD, p<0.05).

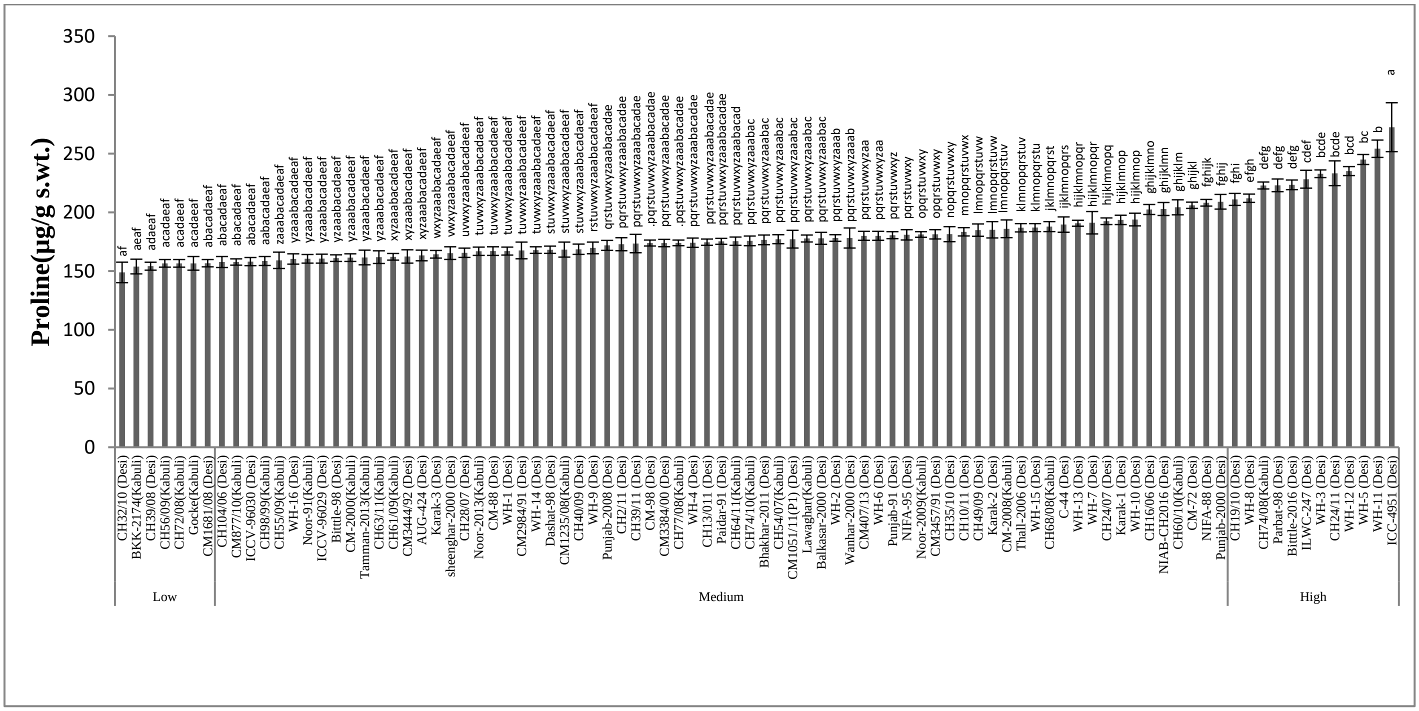

Figure 14: Comparison of seed proline content in chickpea genotypes (mean value ± SD). Means with different alphabets are significantly different (Tukey's HSD, p<0.05).

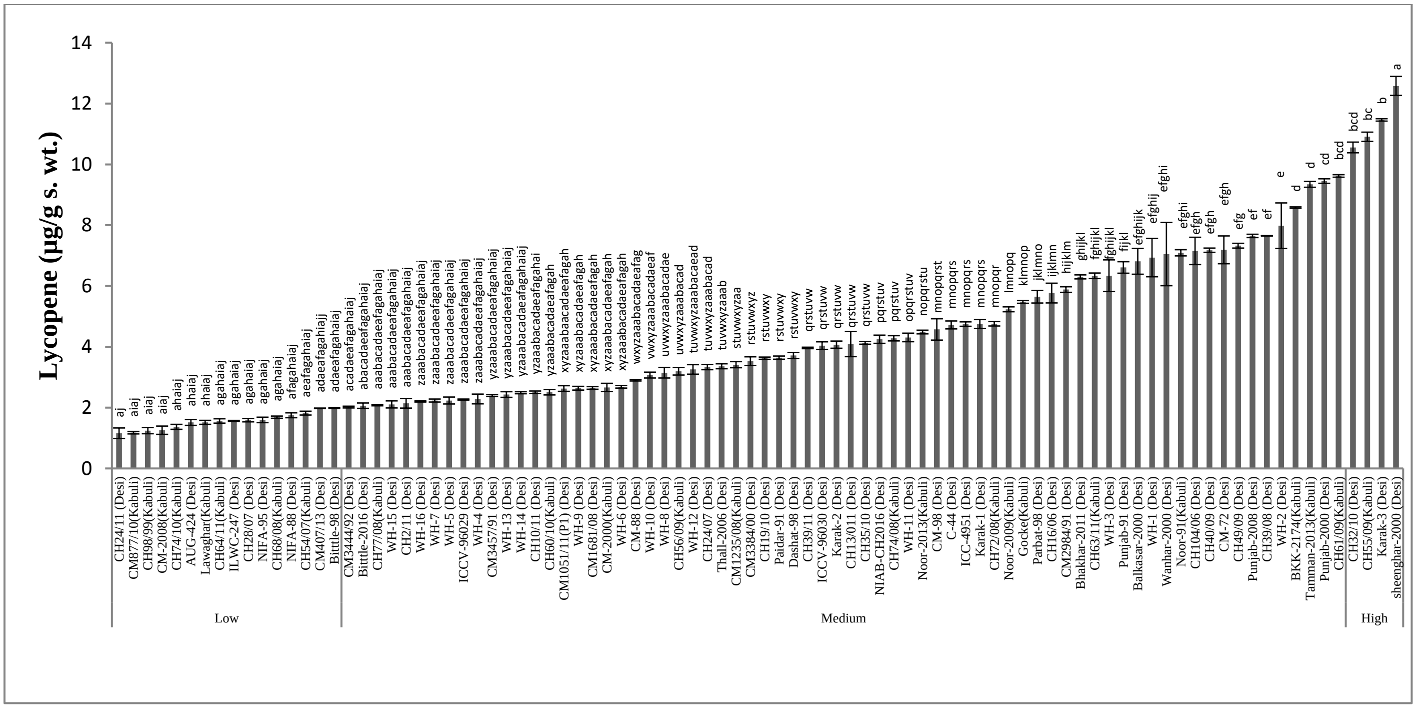

Figure S15: Comparison of seed lycopene content in chickpea genotypes (mean value ± SD). Means with different alphabets are significantly different (Tukey’s HSD, p<0.05).

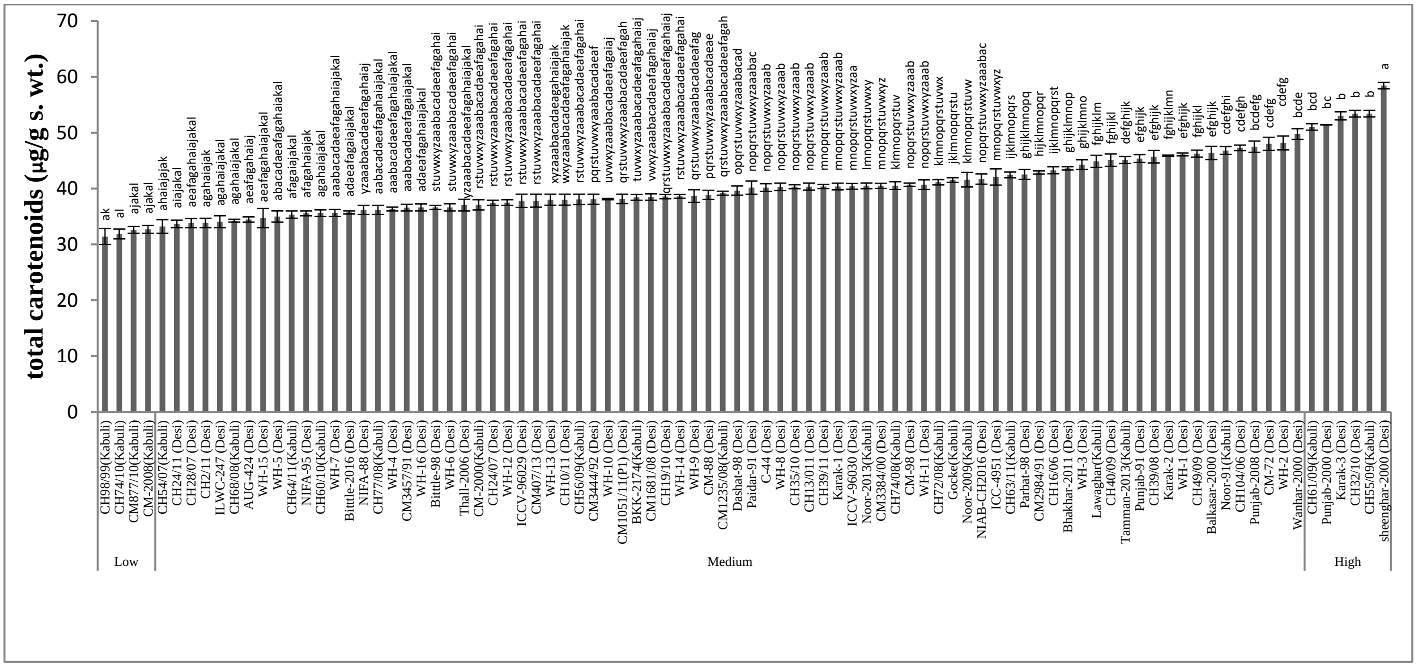

Figure S16: Comparison of seed total carotenoids in chickpea genotypes (mean value ± SD). Means with different alphabets are significantly different (Tukey’s HSD, p<0.05).

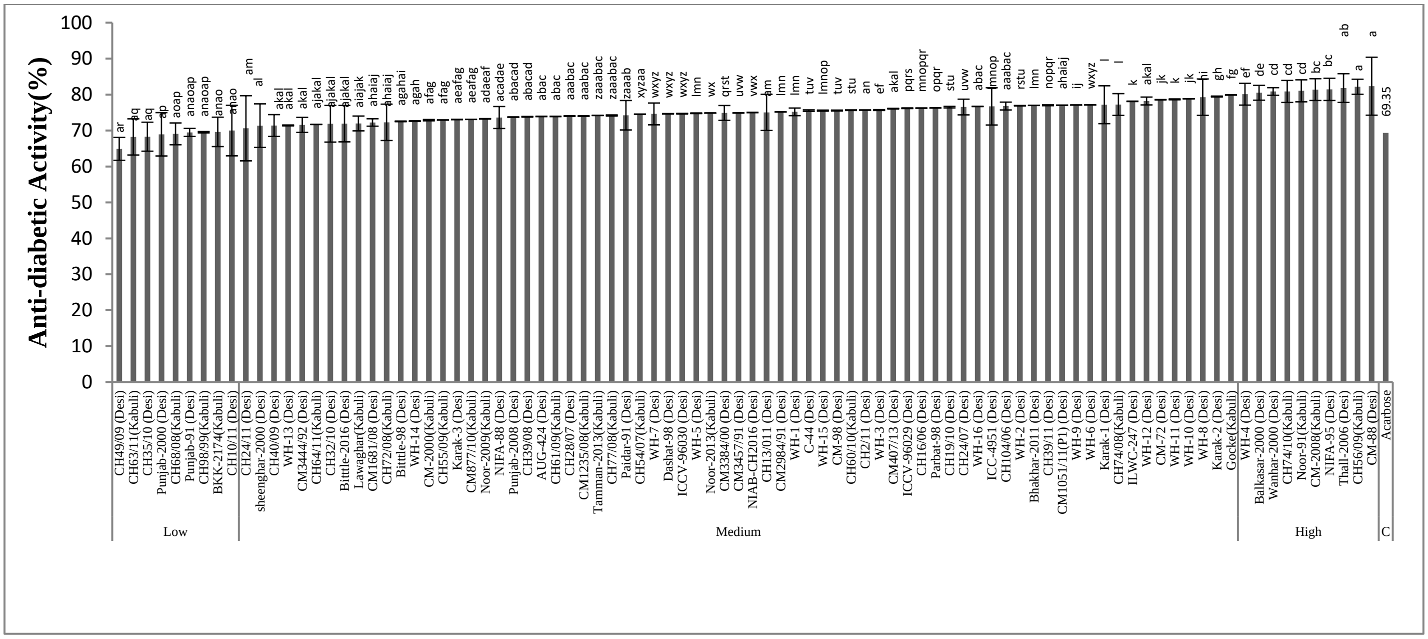

Figure S17: Comparison of seed in vitro anti-diabetic activity in chickpea genotypes (mean value ± SD), Acarboseis standard drug used for comparison. Means with different alphabets are significantly different (Tukey’s HSD, p<0.05).

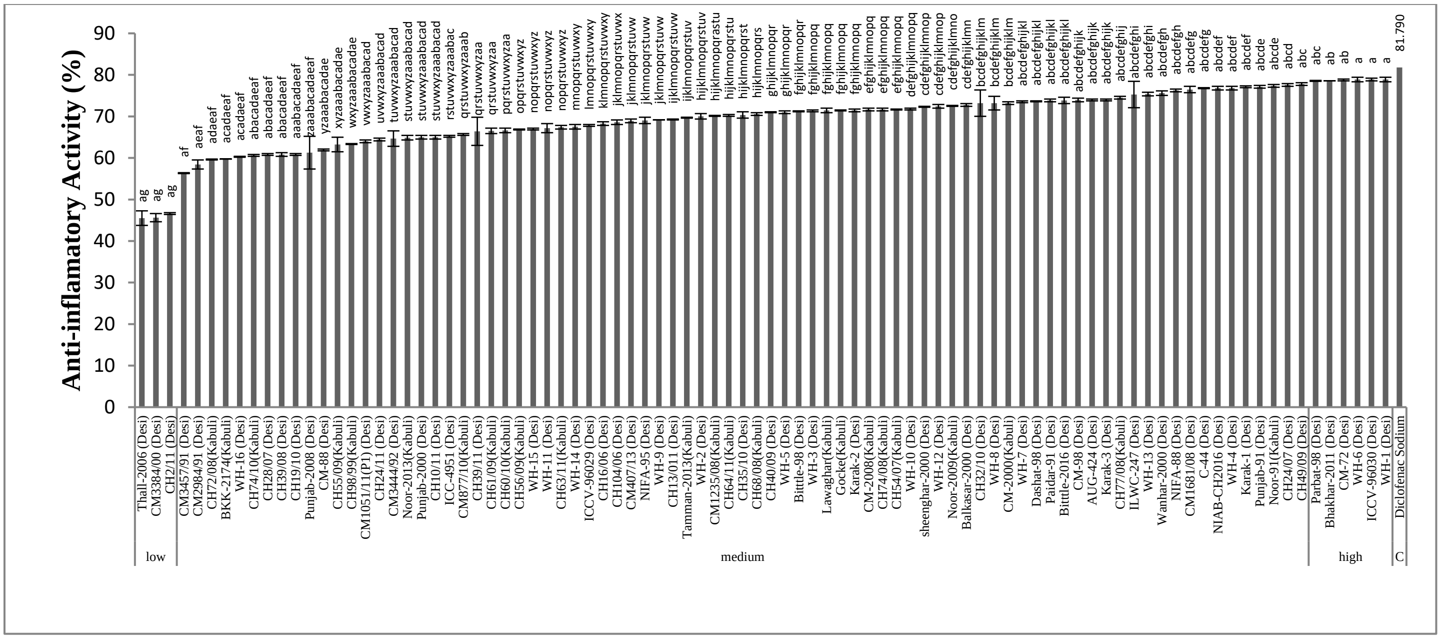

Figure S18: Comparison of seed in vitro anti-inflammatory activity in chickpea genotypes (mean value ± SD), Diclofenac sodium is a standard drug used for comparison. Means with different alphabets are significantly different (Tukey’s HSD, p<0.05).

Table S1: Principal component analysis for different biochemical traits in Chickpea seed flour

**Principal Component Analysis (PCA)**																		
	**F1**	**F2**	**F3**	**F4**	**F5**	**F6**	**F7**	**F8**	**F9**	**F10**	**F11**	**F12**	**F13**	**F14**	**F15**	**F16**	**F17**	**F18**
**Eigenvalue**	3.075	2.419	1.901	1.549	1.308	1.113	1.059	1.016	0.892	0.816	0.679	0.567	0.480	0.409	0.335	0.297	0.054	0.031
Variability (%)	17.082	13.441	10.562	8.604	7.268	6.183	5.885	5.646	4.956	4.535	3.773	3.149	2.668	2.270	1.859	1.653	0.297	0.170
Cumulative %	17.082	30.522	41.084	49.688	56.956	63.139	69.024	74.670	79.626	84.160	87.934	91.083	93.751	96.021	97.880	99.533	99.830	100.000
**Factor loadings**	**F1**	**F2**	**F3**	**F4**	**F5**	**F6**	**F7**	**F8**	**F9**	**F10**	**F11**	**F12**	**F13**	**F14**	**F15**	**F16**	**F17**	**F18**
**TPC**	**0.054**	-0.148	-0.318	**0.251**	0.213	0.517	0.226	-0.112	0.295	0.535	0.043	-0.236	0.034	0.021	-0.046	-0.023	-0.005	0.002
**As.A**	**0.035**	**0.104**	**0.201**	-0.402	0.050	0.558	0.172	0.483	-0.263	-0.177	0.257	-0.111	-0.075	-0.028	-0.078	0.136	-0.011	0.000
**TOS**	-0.599	**0.577**	-0.150	-0.110	-0.392	-0.088	0.266	0.074	0.104	0.067	0.009	0.001	-0.019	-0.004	-0.050	-0.045	0.018	0.122
**Lycopene**	**0.776**	-0.168	-0.403	-0.041	-0.173	-0.123	0.190	0.246	0.002	-0.074	-0.034	-0.068	0.073	0.104	-0.072	-0.064	0.162	-0.008
**TFC**	-0.011	**0.477**	-0.013	**0.067**	0.542	0.109	0.449	-0.056	-0.163	-0.176	-0.248	0.140	0.229	0.235	0.100	-0.034	-0.013	0.003
**Protease**	-0.120	-0.275	**0.663**	**0.305**	-0.245	0.157	-0.041	0.092	-0.150	0.109	-0.300	0.040	-0.120	0.265	-0.250	-0.093	0.003	0.004
**MDA**	-0.565	-0.492	-0.224	**0.080**	-0.064	-0.063	-0.103	0.248	-0.103	0.025	0.304	-0.051	0.006	0.334	0.251	-0.144	-0.015	0.002
**Esterase**	**0.360**	**0.263**	**0.099**	**0.650**	0.028	0.037	0.220	0.183	-0.083	0.046	0.261	0.313	-0.202	-0.182	0.055	-0.182	-0.002	-0.006
**SOD**	-0.468	-0.270	-0.506	**0.444**	0.122	-0.081	-0.007	0.096	-0.014	-0.048	0.073	0.262	0.012	0.061	-0.196	0.324	0.010	0.003
**Proline**	-0.011	-0.382	**0.290**	-0.256	0.349	-0.399	0.409	0.184	0.187	0.171	-0.111	-0.020	-0.350	0.012	0.098	0.116	0.007	0.008
**POD**	**0.038**	-0.393	**0.200**	-0.215	-0.233	0.316	0.177	-0.168	0.585	-0.297	0.095	0.312	0.037	0.072	0.029	-0.017	0.001	-0.004
**Alpha amylase**	**0.374**	-0.109	**0.348**	**0.222**	-0.510	-0.046	0.340	-0.299	-0.253	0.137	0.127	-0.061	0.130	0.046	0.179	0.241	-0.004	0.002
**TAC**	**0.615**	-0.565	**0.154**	**0.086**	0.371	0.100	-0.277	-0.083	-0.104	-0.074	0.038	0.035	0.057	-0.033	0.040	0.012	0.005	0.123
**APX**	-0.168	-0.419	-0.370	**0.021**	-0.004	0.022	0.344	-0.478	-0.258	-0.350	0.047	-0.186	-0.234	-0.041	-0.125	-0.118	-0.022	0.002
**CAT**	-0.380	-0.521	**0.266**	-0.252	0.112	-0.229	0.267	0.052	-0.144	0.201	0.164	0.111	0.362	-0.186	-0.169	-0.130	0.001	-0.009
**Total Carotenoids**	**0.787**	-0.076	-0.362	-0.074	-0.211	-0.221	0.119	0.262	0.088	0.003	-0.046	-0.005	0.067	0.090	-0.116	-0.027	-0.160	0.008
**Anti inflamatory activity**	**0.331**	**0.346**	-0.082	-0.503	0.111	-0.051	-0.121	-0.357	-0.159	0.304	0.288	0.257	-0.144	0.227	-0.122	-0.007	0.012	-0.003
**A. diabetic activity**	**0.080**	**0.367**	**0.452**	**0.309**	0.246	-0.297	-0.002	-0.057	0.294	-0.211	0.373	-0.298	0.071	0.134	-0.159	0.018	0.004	0.000
